# eCOMPASS: evaluative comparison of multiple protein alignments by statistical score

**DOI:** 10.1093/bioinformatics/btab374

**Published:** 2021-05-13

**Authors:** Andrew F Neuwald, Bryan D Kolaczkowski, Stephen F Altschul

**Affiliations:** Department of Biochemistry and Molecular Biology, University of Maryland School of Medicine, Baltimore, MD 21201, USA; Department of Microbiology and Cell Science, University of Florida, Gainesville, FL 32611, USA; Computational Biology Branch, National Center for Biotechnology Information, National Library of Medicine, National Institutes of Health, Bethesda, MD 20894, USA

## Abstract

**Motivation:**

Detecting subtle biologically relevant patterns in protein sequences often requires the construction of a large and accurate multiple sequence alignment (MSA). Methods for constructing MSAs are usually evaluated using benchmark alignments, which, however, typically contain very few sequences and are therefore inappropriate when dealing with large numbers of proteins.

**Results:**

eCOMPASS addresses this problem using a statistical measure of relative alignment quality based on direct coupling analysis (DCA): to maintain protein structural integrity over evolutionary time, substitutions at one residue position typically result in compensating substitutions at other positions. eCOMPASS computes the statistical significance of the congruence between high scoring directly coupled pairs and 3D contacts in corresponding structures, which depends upon properly aligned homologous residues. We illustrate eCOMPASS using both simulated and real MSAs.

**Availability and implementation:**

The eCOMPASS executable, C++ open source code and input data sets are available at https://www.igs.umaryland.edu/labs/neuwald/software/compass

**Supplementary information:**

Supplementary data are available at *Bioinformatics* online.

## 1 Introduction

Protein sequence analyses, and particularly those that are statistically based, often rely upon very large multiple sequence alignments (MSAs), consisting of tens or hundreds of thousands of sequences belonging to a large superfamily. Using such an alignment increases the statistical power and breadth of an analysis and, by partitioning the MSA into hierarchically arranged subgroups based on subgroup-specific patterns (Neuwald, 2014), one can identify sequence and structural features likely determining functional specificity. For example, this approach has been used (Neuwald *et al.*, 2012) to automate the manual curation of hierarchical MSAs (hiMSAs) for the NCBI Conserved Domain Database (CDD) (Yang *et al.*, 2020) and, when applied to an MSA of 474 040 AAA+ ATPases, has revealed sequence and structural properties implicated in DNA clamp loader functional specificity (Tondnevis *et al.*, 2020). We have performed similar analyses using alignments of 237 359 N-acetyltransferases, 127 418 GTPases, 131 321 helicases, 45 799 exonuclease-endonuclease-phosphatases and 23 592 DNA glycosylases (Neuwald *et al.*, 2018) and of 33 760 TIR domains (Toshchakov and Neuwald, 2020). It is important, of course, that such alignments be as biologically accurate as possible. However, it is well known that only heuristic methods are available for constructing even small alignments, and these produce results that may be far from optimal (Edgar, 2010). Generally, an MSA method’s accuracy is evaluated using a set of benchmark alignments that are manually curated using structural data, and which typically contains relatively few sequences. However, there are many potential problems with these evaluations. First, they rely upon the accuracy of the benchmark alignments, which may itself be in question (Ashkenazy *et al.*, 2019; Fletcher and Yang, 2010; Kim and Lee, 2007; Levy Karin *et al.*, 2014; Thompson *et al.*, 2011). Second, they implicitly assume the accuracy of an MSA on a benchmark set of sequences is a good proxy for its accuracy on a much larger superset. This may not be the case, particularly when the larger set contains many protein subgroups within a superfamily, not all of which are represented within the benchmark alignment. Curating large benchmark MSAs is error prone and may be prohibitively labor intensive. Finally, the relative accuracy of one MSA method to another on a set of benchmark alignments is no guarantee that it will produce the more accurate alignment for a specific set of sequences of interest, particularly one that is large and diverse.

We define an accurate alignment to be one that reflects sequence homology. A more accurate MSA should reveal evolutionarily conserved structural and functional constraints better than a less accurate one. In large, diverse sequence sets such constraints become more statistically evident, thereby allowing subtly conserved homologous regions to be identified and aligned, as illustrated in Neuwald and Hirano (2000) and Neuwald and Poleksic (2000).

Because obtaining a highly accurate MSA typically requires manual curation, we have developed and applied the Multiply Aligned Profiles for Global Alignment of Protein Sequences (MAPGAPS) program (Neuwald, 2009), which uses a manually curated hiMSA as a query to identify and align database sequences belonging to a modeled superfamily. Within a hiMSA each subgroup alignment is profiled and aligned to the other subgroup alignments. Using this feature, MAPGAPS creates an MSA with accuracy comparable to that of the hiMSA (Neuwald *et al.*, 2020). This assumes that each subgroup is accurately aligned both internally and relative to other subgroups, which is typically not yet the case. Hence, to further improve this approach, we need to assess alignment quality for each subgroup and for the MSA as a whole.

Here, we introduce eCOMPASS, a program that evaluates the relative accuracy of two MSAs of the same large set of sequences by applying direct coupling analysis (DCA) based upon pseudo-likelihood maximization in conjunction with a procedure to estimate statistical significance. It requires as input only the MSAs themselves and structural coordinates for a minimum number (ideally at least 10) of the aligned sequences. It does not rely upon any set of benchmark alignments, nor even upon a *‘*gold standard*’* alignment of the subset of sequences with known structure. Furthermore, it requires no knowledge of how the MSAs were produced, nor upon how the methods that produced them perform on other sets of sequences. Rather, for each MSA, it first derives, from pairwise correlations among columns, internal evidence of likely 3D contacts among residue positions of the aligned proteins, and then uses the known structures to assess the relative accuracy of this evidence. This approach is based on the principle that, to maintain a protein family’s structural fold, interacting residues pairs tend to coevolve, resulting in correlations better seen within accurate alignments. Hence, the degree to which 3D contacts may be correctly inferred from an MSA depends upon its accuracy.

Because eCOMPASS applies to the evaluation of the overall quality of specific sequence alignments that are very large, it cannot be readily evaluated using known benchmark MSAs, nor are we aware of previous approaches to which it can be properly compared. We therefore argue for its validity from its inherent plausibility, its application to simulated gold standard alignments, and its consistency with a completely independent measure of alignment accuracy than the measure eCOMPASS deploys.

We first describe the eCOMPASS algorithm and illustrate its use by applying it to eight pairs of large MSAs obtained from the CDD and PFAM databases and containing a sufficient number of proteins of known structure. We also describe the sort of insights eCOMPASS can reveal regarding the relative quality of such MSAs. Second, we validate it on simulated MSAs generated from realistic Potts models of protein superfamilies versus realignments of the simulated sequences using four different alignment methods. Third, we evaluate its robustness to changes in various hyperparameter settings.

## 2 Materials and methods

### 2.1 Input and basic strategy

eCOMPASS takes as input two MSAs of the same set of protein sequences aligned using two different methods. We recommend that the set include at least 10 proteins of known structure. The method’s basic strategy is, first, to use correlations among columns in each MSA to predict which pairs of columns correspond to residue 3D contacts; and then to check the accuracy of these predictions (measured as described below) using the aligned proteins of known structure. The method assumes that the more accurate the overall MSA, the more accurate will be structural predictions derived from its column correlations. Evidence for the validity of this assumption is provided through analyses of simulated MSAs.

Note that, although eCOMPASS uses a relatively small number of sequences with known structure to vote on the relative accuracy of two MSAs, each structure’s vote is based upon evidence derived from all the sequences in each of the MSAs. Thus, an MSA that accurately aligns the structures in question to one another but does a poor job of aligning sequences from a much larger and more diverse protein superfamily, should fare poorly in eCOMPASS’s estimation. This contrasts with evaluation methods that use the accurate alignment of a (typically small) test set alone as a proxy for an MSA’s more general accuracy. Note also that eCOMPASS requires no *‘*gold standard*’* alignments whose accuracy must be assumed. It bases its evaluation only on the given MSAs and on the experimentally determined structures.

### 2.2 DCA

In order to infer structural information from correlations between column pairs of each MSA, as a prelude to assessing the accuracy of this information, eCOMPASS first performs on the alignments DCA (Hopf *et al.*, 2012; Lunt *et al.*, 2010; Morcos *et al.*, 2011; Nugent and Jones, 2012; Weigt *et al.*, 2009). Residue pairwise correlations were long believed, in principle, to be predictive of structural contacts, but early approaches fell short of expectations due to the confounding effect of indirect correlations: when residues correlate both at positions *i* and *j* and at positions *j* and *k*, then residues at positions *i* and *k* may also correlate even though they fail to interact directly. DCA overcomes this problem by disentangling direct from indirect correlations using a variety of algorithmic strategies. eCOMPASS uses pseudo-likelihood maximum entropy optimization (Marks *et al.*, 2011, 2012) as implemented in CCMpred (Seemayer *et al.*, 2014); this strategy outperformed (Neuwald and Altschul, 2018) DCA programs based either on sparse inverse covariance estimation (Jones *et al.*, 2012) or on multivariate Gaussian modeling (Baldassi *et al.*, 2014).

Many multiple alignment methods construct an idealized model to which individual protein sequences are aligned, resulting in some residues being treated as insertions with respect to this model, and therefore left essentially unaligned to residues in other sequences. For an MSA constructed by such a method, it is only the columns corresponding to modeled positions to which we apply DCA, and we effectively ignore all inserted residues. Other multiple alignment methods align all residues in all input sequences, but this usually results in many columns having null characters for most sequences. To apply DCA effectively to such alignments, we first exclude columns having greater than 50% null characters.

The output of DCA applied to an MSA *M*_1_ is a set *K*_1_ of direct coupling (DC) scores for all of *M*_1_’s column pairs. DC scores correspond to the average product corrected Frobenius norms (Dunn *et al.*, 2008; Seemayer *et al.*, 2014). (DCA methods model both one- and two-site statistics, though eCOMPASS makes use of only the latter.) We assume only that these scores grow monotonically with the degree of inferred DC between MSA columns. We observe, however, that there is no immediate way to compare the set *K*_1_ with an analogous set *K*_2_ derived from *M*_2_, both because they typically will differ in size, and because there is no clear correspondence between the columns of *M*_1_ and *M*_2_.

We address this issue by using the sequence of each protein with known structure, considered individually, to choose comparable subsets of *K*_1_ and *K*_2_, which we call $K_{1}^{ʹ}$ and $K_{2}^{ʹ}$. Specifically, for a given protein, we first determine the subset *R* of its residues that are aligned both in a column in *M*_1_ and in a column in *M*_2_. Identifying the residues in *R* with the MSA columns to which they are aligned, we define $K_{1}^{ʹ}$ (and $K_{2}^{ʹ}$ analogously) as the subset of *K*_1_ corresponding to all pairs of residues in *R* separated by at least *m* (5 by default) intervening residues within the protein’s primary sequence. (We impose this latter condition because we are not interested in predicting close contacts that are imposed by a protein’s backbone.) $K_{1}^{ʹ}$ and $K_{2}^{ʹ}$ are then of equal size, with elements corresponding to identical pairs of residues within *R*. Note, however, that each individual structure defines distinct $K_{1}^{ʹ}$ and $K_{2}^{ʹ}$, and it is only such sets, constructed from the same structure, that are directly comparable.

### 2.3 Initial Cluster Analysis

Our approach is based on the assumptions that within a protein family the evolution of structurally interacting residue pairs is likely to be correlated, and that an accurate multiple alignment of sequences in the family should capture information concerning such correlations in the form of high DC scores. Given two MSAs for a protein family, and a particular structure, we have constructed sets of DC scores, $K_{1}^{ʹ}\;$and $K_{2}^{ʹ}$, each of whose elements correspond to the same set of residue pairs of known 3D distance and are therefore comparable. We note, however, that inherent differences, such as differing numbers of columns, in the MSAs *M*_1_ and *M*_2_ that are used to construct first *K*_1_ and *K*_2_, and then $K_{1}^{ʹ}$ and $K_{2}^{ʹ}$, renders problematic the direct comparison of the raw scores within $K_{1}^{ʹ}$ and $K_{2}^{ʹ}$. Instead, we assume only that higher scores within each set should be preferentially associated with closer structural distances.

To measure the strength of the association between DC scores and physical distances, we turn to Initial Cluster Analysis (ICA) (Altschul and Neuwald, 2018). ICA considers an ordered array of *L* elements, among which *D* are designated as distinguished, and seeks the initial segment of the array, of length *X*, with the most surprising number *d* of distinguished elements, as measured by a *P*-value. A generalization of ICA that has been applied to DC scores (Neuwald and Altschul, 2018), and which we employ here, adds an ordering to the distinguished elements, and folds into its optimization a statistical measure of the degree to which the higher ranked among the distinguished elements appear earlier in the array. In essence, this generalization can be understood as measuring the degree of congruence between two ordered sets.

Here, we take the array of elements to be the set of DC scores $K_{1}^{ʹ}$ (or $K_{2}^{ʹ}$), ordered from highest to lowest. The distinguished elements are those corresponding to residue pairs whose structural distance is **≤***z* (with *z* = 4 Å by default). Note that, except for glycine, *z* is based on the distance between sidechain atoms rather than between *α*- or β-carbons. ICA returns an *S*-score (Neuwald and Altschul, 2018) calculated as $S=-\log_{10}\left(P\right)$. *S*-scores have units of log-probability and are therefore directly comparable. Nevertheless, when the relationship between two orderings is known, or strongly suspected, to be significant, an array with a larger number of elements *L*, and/or a larger number of distinguished elements *D*, may intrinsically favor the generation of higher or lower *S*-scores. In such cases, it is best to compare only *S*-scores generated from arrays with the same *L* and *D*. Because the scores *S*_1_ and *S*_2_ we calculate for our two input MSAs from $K_{1}^{ʹ}$ and $K_{2}^{ʹ}$ are, by construction, generated using the same *L* and *D*, we take their difference ΔS = *S*_1_ − *S*_2_ as a valid measure of the evidence provided by the structure in question for the relative accuracies of MSAs *M*_1_ and *M*_2_. In this study, an *S*-score can be understood as a statistical measure of the congruence of structural contacts with DC scores (i.e. average product corrected Frobenius norms).

### 2.4 Eliminating structures likely to be misaligned

It would be possible to assess the relative quality of *M*_1_ and *M*_2_ by evaluating solely how well each MSA aligns the reference structures to one another. However, this would ignore how the vast number of remaining sequences are aligned. In contrast, eCOMPASS measures how well the DC scores derived from each MSA predict 3D contacts between residue pairs in each reference structure. This assumes, however, that each structure is properly aligned, in the main, within both MSAs, which may not be the case.

To identify reference structures that may be misaligned within a particular MSA, we first determine, for each structure *i*, the subset *R_i_* of its residues that are aligned by the MSA to residues rather than null characters in all other structures; note that the *R_i_* will be of the same size for all structures. We then compute, for each pair of structures *i* and *j*, the quantity Δ𝔇_*ij*_, defined as the mean, for all pairs of residues *a* and *b* within *R_i_*, of the absolute difference between the Cα distance of *a* to *b* and the Cα distance within structure *j* of the residues to which *a* and *b* align. It can be seen that Δ𝔇_*ij*_ = Δ𝔇_*ji*_, and this quantity may be understood to measure how well sequences *i* and *j* are structurally aligned with one another (Hasegawa and Holm, 2009; Holm *et al.*, 2008). Assuming most structures are on average properly aligned, a structure *i* that is poorly aligned should have high Δ𝔇_*ij*_ for most *j*, and therefore an unusually high mean value of Δ𝔇_*ij*_ for all *j* ≠ *i*, which we denote as Δ𝔇_*i*_. Any structure whose Δ𝔇_*i*_ is ≥2 SD above the mean is likely to be misaligned and thus to yield unreliable results, and we accordingly may choose to remove it from consideration. We iteratively recalculate until convergence the mean and SD from the remaining Δ𝔇_*i*_, and each time remove any structure whose Δ𝔇_*i*_ is ≥2 SD above the mean. Of course, to apply this approach effectively it is important to have a sufficient number of diverse structures (corresponding by default to proteins sharing ≤65% sequence identity). After all structures with questionable alignment within either MSA have been removed, we calculate $\bar{\Delta S}$, the mean value of ΔS, both for the remaining structures and for all structures, as two alternative measures of the relative quality of *M*_1_ and *M*_2_.

Note that the number of columns used to calculate the Δ𝔇_*i*_ varies from one MSA to another, as of course do the subsets of residues *R_i_* within the various structures. Thus, in contrast to the *S_i_*, the Δ𝔇_*i*_ are properly comparable only among different structures for the *same* MSA, but not between one MSA and another. Nevertheless, as we will see below, there is a noticeable tendency for the MSA preferred by the measure $\bar{\Delta S}$ also to yield a lower $\bar{\Delta D}$ (mean Δ𝔇_*i*_), which can be understood as a rough measure of how well an MSA aligns the reference structures to one another.

### 2.5 Using simulated Potts model MSAs as gold standards

We created a Potts model for each of 40 CDD/MAPGAPS-generated MSAs (listed in Supplementary Table S1) using CCMpredPy (Vorberg *et al.*, 2018). To obtain 3D contacts for each Potts model, we created corresponding homology modeled structural coordinates using SWISS-MODEL (Waterhouse *et al.*, 2018); column pairs corresponding to 3D contacts >8 Å in the structure are set to zero in the Potts model generated by CCMpredPy. A simulated 5000 sequence alignment was generated for each Potts model using CCMgen (Vorberg *et al.*, 2018). We realigned the sequences for each of the simulated MSAs using four different MSA programs (see below) and used eCOMPASS to score each realigned MSA when compared to the corresponding gold standard MSA.

## 3 Application

### 3.1 Overview

Most commonly used multiple alignment programs fail to generate plausible MSAs when given as input the numbers of sequences considered in this study, typically in the tens or hundreds of thousands. Therefore, we do not attempt to evaluate these programs, but instead apply eCOMPASS in three ways: (i) to 8 CDD versus PFam MSAs; (ii) to 40 realigned versus gold standard simulated MSAs; and (iii) to 31 CDD versus JackHMMER MSAs using various eCOMPASS hyperparameter settings.

### 3.2 CDD versus Pfam MSAs

We illustrate eCOMPASS using eight pairs of MSAs (Table 1), each consisting of one CDD-based MSA (obtained as described in Table 1) and one Pfam MSA (El-Gebali *et al.*, 2019). These MSA pairs represent the following protein superfamilies: C2 domains (C2); cupredoxins (CuDX); haloacid dehalogenase-like hydrolases (HAD); class B metal β-lactamases (MBL); pleckstrin homology domains (PH); phosphotransferase system subunit IIB (PTS); rhodanese homology domain (RHOD) and sulfatases (SFTS). We obtained a mean of 25 reference structures per domain. Over their domain footprints, on average these share 19% sequence identity, and each structure shares <50% identity with all other structures. Thus, these represent well the diversity of each superfamily. The eCOMPASS output files are available as Supplementary Material. ‘CDD’ MSAs achieved, on average, higher *S*-scores than Pfam MSAs (Table 2). However, because both types of alignments depend on some degree of manual curation, we draw no general conclusion regarding which of these tend to be more accurate. Rather, our aim here is merely to describe eCOMPASS and illustrate its application.

**Table 1.** Eight pairs of CDD versus Pfam MSAs analyzed here

Name		MSA1		MSA2			Avg
Abbr.	# seqs	Len	CDD	Len	Pfam	#pdb	%id
C2	72 249	102	cd00030	103	PF00168	34	22
CuDX	15 418	110	cd00920	119	PF07732	20	23
HAD	58 031	95	cd01427	95	PF00702	18	21
MBL	70 293	188	cd06262	197	PF00753	32	14
PH	36 099	89	cd00900	105	PF00169	30	17
PTS	9395	84	cd00133	90	PF02302	13	18
RHOD	61 053	89	cd00158	107	PF00581	33	19
SFTS	35 560	237	cd00016	309	PF00884	21	19
Mean	44 762	124		141		25	19

The numbers of aligned sequences for each domain are given in column 2. Lengths of MSA 1 and 2 are given in columns 3 and 5, respectively, and corresponding CDD and Pfam identifiers are given in columns 4 and 6, respectively. CDD alignments were obtained using, as input to MAPGAPS, the NCBI CDD hiMSA and the sequences present in the corresponding Pfam MSA, as was recently described (Neuwald *et al.*, 2020). Each Pfam MSA had been generated automatically by creating a hidden Markov model profile from a Pfam seed alignment and then aligning related sequences to the profile (Sonnhammer* et al*., 1998). For each analysis, the number of reference structures and the average % identity shared among aligned regions of known structure are given in columns 7 and 8, respectively.

**Table 2.** eCOMPASS results with outliers excluded

ID	MSA 1		MSA 2		$\bar{\Delta S}$	SD	−log_10_(*P*)
	*N*_1_	$\bar{\Delta D}$	*N*_2_	$\bar{\Delta D}$			
C2	0	1.12	25	1.15	−9.7	5.3	7.2
CuDX	12	1.19	4	0.89	2.4	5.3	1.1
HAD	4	1.27	10	1.18	−4.3	8.9	0.7
MBL	18	1.74	0	3.28	82.1	16.5	5.1
PH	20	0.99	0	1.43	8.9	6.4	5.7
PTS	12	2.19	0	2.89	11.2	5.6	3.3
RHOD	19	1.57	9	2.18	6.5	10.3	1.1
SFTS	14	1.40	5	1.80	15.5	19.7	1.2

For each domain, values of $\bar{\Delta D}$ and $\bar{\Delta S}$ were calculated only after excluding unreliably aligned structures, as described in the text. *N*_1_ and *N*_2_ are the observed number of included structures for which *S*_1_ > *S*_2_ and *S*_2_ > *S*_1_, respectively. The Δ*S*-score standard deviation (SD) measures the variability among reference structures for each domain. For the last column, *P* is calculated as the two-tailed binomial probability for the observed *N*_1_ and *N*_2_, assuming an equal chance for each MSA to have higher ΔS for each structure.

### 3.3 CDD versus Pfam subgroup-specific analyses

Because a protein superfamily is typically composed of multiple families and subfamilies, which may be aligned with differing accuracy, the ΔS scores for different structures should not be considered as drawn from the same underlying distribution and their variance may therefore be very high. Accordingly, when asking which is the more accurate of two MSAs overall, it is better to consider each ΔS score as a separate vote. Assuming independence for simplicity, we calculate the significance of the majority vote using the two-tailed *P*-value for the equiprobable binomial distribution. We expect these *P*-values to correlate to some extent with $\bar{\Delta S}$, the mean ΔS score, but these two quantities may vary considerably in implied significance, or, in principle, even disagree on which is the better MSA. Also, we recognize that even two structures with low sequence identity are not truly independent, so that our calculated *P*-values must be discounted to some extent.

In Table 2, we present a summary of eCOMPASS’s results for the eight domains considered. After putatively misaligned reference structures are excluded, for four domains (C2, MBL, PH and PTS) eCOMPASS finds unanimity among the remaining structures favoring one of the MSAs. These agreements are statistically significant, with the Pfam MSA favored for C2 and the CDD MSA favored for MBL, PH and PTS. (This frequent unanimity is evidence that the ΔS score is no mere random artifact but is a valid measure for the greater ability of one MSA to encode structural features as directly coupled residue pairs.) For the remaining four domains, neither MSA is preferred with an estimated *P* < 0.001, and the SD of the ΔS values exceeds their absolute mean.

To illustrate and study our procedure for excluding structures, we consider in detail its operation on the PTS domain. In Table 3, we show the specific values of *S* and Δ𝔇_*i*_ for each of the 13 reference structures and each MSA. As is apparent, only for structure 3czcA and MSA 1 does Δ𝔇_*i*_ exceed the mean by over two SDs, so we exclude this one structure as unreliably aligned. (When the mean and SD for the remaining Δ𝔇_*i*_ for MSA 1 are recalculated, no further structures are excluded.) Note that this has the effect of eliminating the one negative ΔS, leaving unanimous preference for MSA 1 among the remaining structures. An examination of the structures eliminated by our procedure for the other seven domains shows that they very often yield outlying values of ΔS, although this is neither expected nor observed to be universally the case.

**Table 3.** eCOMPASS output for the PTS domain

pdbid	MSA 1		MSA 2		ΔS	cols	*D*	*L*
	*S*_1_	Δ𝔇_*i*_	*S*_2_	Δ𝔇_*i*_				
**3czcA**	**29**	2.78	**41**	2.94	**−12.0**	**82**	**100**	**2944**
2wy2D	47	2.10	34	2.60	12.9	77	85	2583
2l2qA	21	2.42	15	3.00	5.9	65	39	1801
4mgeA	51	2.06	38	2.48	12.7	78	93	2659
3nbmA	54	2.24	30	2.87	23.5	76	88	2525
1tvmA	29	2.34	19	2.92	10.2	74	60	2367
5gqsA	31	2.23	15	2.92	15.8	78	79	2647
1vkrA	28	2.12	11	3.07	16.7	71	64	2164
5dleA	32	2.08	24	2.95	7.3	77	97	2590
2r48A	32	2.11	22	2.91	10.1	77	93	2590
4tn5A	24	2.12	16	2.90	7.5	75	86	2453
2kyrA	22	2.37	20	3.07	2.4	77	90	2595
2m1zA	31	2.10	22	2.92	9.0	77	87	2594
Mean		2.24		2.89	9.4			
SD		0.20		0.17	8.4			

Values for 3czcA are shown in bold to indicate that its Δ𝔇 value for MSA 1 is ≥2 SD above the mean. The 7th column gives the number of columns shared by MSA 1 and 2 when computing *S*-scores. Columns 8 and 9 give the values of *D* and *L* for the ICA procedure.

**Fig. 1.**
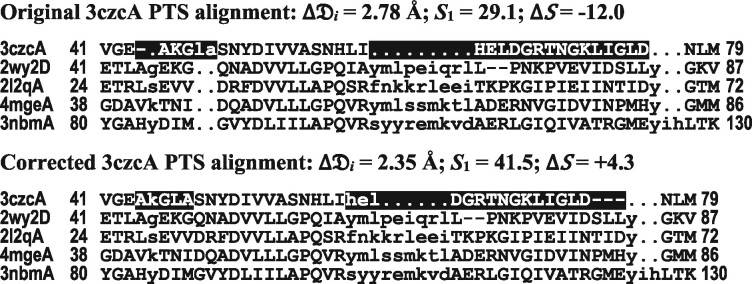
Δ𝔇_*i*_ ≥ 2 SD above the mean for 3czcA is due to misalignment. (**top**) For the CDD PTS MSA, the sequence corresponding to 3czcA yielded Δ𝔇_*i*_ = 2.78 Å, which is 2.7 SD above the mean, suggesting this structure is misaligned relative to the 12 other structures, four of which are shown. As a result, eCOMPASS discarded 3czcA’s ΔS value when computing $\bar{\Delta S}$ =  11.2 in Table 2. (**bottom**) When 3czcA was structurally realigned using Dali (Holm and Rosenström, 2010), its Δ𝔇_*i*_ decreased to 2.35 Å (1.5 SD above the mean) and its *S*-score increased to 41.5, providing further evidence that it was originally misaligned. The realigned region is highlighted in black; numbers correspond to the residue positions at each end.

It is not eCOMPASS’s function to amend the MSAs with which it is supplied. However, to study further the validity of eCOMPASS’s procedure for rejecting structures as misaligned, and their corresponding ΔS as unreliable, we used Dali (Holm and Rosenström, 2010) to structurally realign 3czcA to the other structures. As shown in Figure 1, given the resulting modified MSA 1, Δ𝔇_*i*_ for 3czcA is no longer an outlier, and the $\bar{\Delta S}$ for 3czcA turns positive. Note, however, that sequences closely related to 3czcA in MSA 1 were not realigned; if they had been, presumably the ΔS would have increased further.

One may object to our procedure for excluding a structure, from one or both MSAs, based upon internal evidence that it has been misaligned. Such a structure generally represents not only itself but also the alignment of closely related sequences, and arguably should have a vote equal to that of other structures regarding which alignment is better. In Table 4, we give the results of our analysis if no structures are excluded. As might be expected, the values of $\bar{\Delta D}$ in Table 4 are higher, although this need not always be the case because the removal of a structure due to a significantly high Δ𝔇_*i*_ for one MSA may decrease $\bar{\Delta D}$ for the other MSA. Also, for all domains except CuDX, the standard deviation of the ΔS is higher. This too is expected, because, although structures are removed with no reference to ΔS, misaligned structures have a strong tendency to produce outlying values for ΔS, as illustrated, e.g. in Table 3. Most importantly, however, for all domains the assessment of which is the better MSA is essentially unchanged, by the measure either of $\bar{\Delta S}$ or of the binomial vote *N*_1_ versus *N*_2_. There appears to be a slight tendency for both $\left|\bar{\Delta S}\right|$ and −log_10_(*P*) to decrease with the inclusion of all structures, but this is neither systematic nor coordinated. The advantage of excluding apparently misaligned structures is that this focuses more on the overall quality of the MSAs, as measured by their DC signal, and less on the alignment accuracy of the relatively small number of structures considered. To help assess such distinctions, eCOMPASS computes results using both approaches.

**Table 4.** eCOMPASS results with outliers included

ID	MSA 1		MSA 2		$\bar{\Delta S}$	SD	*−*log_10_(*P*)
	*N*_1_	$\bar{\Delta D}$	*N*_2_	$\bar{\Delta D}$			
C2	3	1.43	31	1.53	−8.1	6.2	6.1
CuDX	15	1.19	5	0.96	2.1	5.1	1.4
HAD	6	1.35	12	1.33	−4.3	8.9	0.6
MBL	31	2.06	1	3.79	74.0	28.0	7.8
PH	29	1.12	2	1.59	9.4	14.3	6.3
PTS	12	2.24	1	2.89	9.4	8.4	2.5
RHOD	24	1.71	9	2.32	6.4	9.6	1.9
SFTS	15	1.52	6	1.84	13.0	24.4	1.1

For some superfamilies neither MSA was significantly favored based on the binomial *P*-value. For example, for the sulfatases (SFTS) *P* = 0.06 and, among the retained ΔS scores, 14 were positive (favoring MSA 1) and 5 were negative (favoring MSA 2). The variability in ΔS scores was very high with an SD of 19.7 and a mean of 15.5. Similar results were obtained when using all ΔS scores. This suggests that MSA 1 better aligns some functionally divergent subgroups while MSA 2 better aligns others. This may occur, e.g. when an MSA is generated by a query-based iterative alignment method, such as PSI-BLAST (Altschul* et al*., 1997) or JackHMMER (Johnson *et al.*, 2010), resulting in subgroups closely related to the query being more accurately aligned than distantly related subgroups. The Pfam MSAs used for this study were generated using a similar profile-based alignment method.

By providing a more articulated description of relative alignment quality than would a single measure of overall quality, eCOMPASS may aid the curation of hiMSAs (Yang *et al.*, 2020), which were provided as input to MAPGAPS to generate the MSA 1 alignments used here. For instance, for the SFTS domain, the structure 4uplA, which is a member of the phosphonate monoester hydrolase family (i.e. cd16028), has the lowest ΔS score (−53.2) and the highest Δ𝔇_*i*_ (2.99 Å) for MSA 1 (see Supplementary Data). This suggests that, by further curating the cd16028 subgroup, one could improve the CDD hiMSA and thus the SFTS MSA generated from it.

Finally, as discussed above, $\bar{\Delta D}$ scores should only be compared with caution because both the numbers and the nature of the residue pairs used to compute Cα–Cα distances differ between MSAs. For example, unlike other domains, the C2 MSA deemed superior by the measure of $\bar{\Delta S}$ (Tables 2 and 4) yielded higher $\bar{\Delta D}$. This illustrates how relying on $\bar{\Delta D}$ scores may miss distinctions between MSAs revealed by better justified and statistically based $\bar{\Delta S}$ scores.

### 3.4 Program-aligned versus gold standard simulated MSAs

**Fig. 2.**
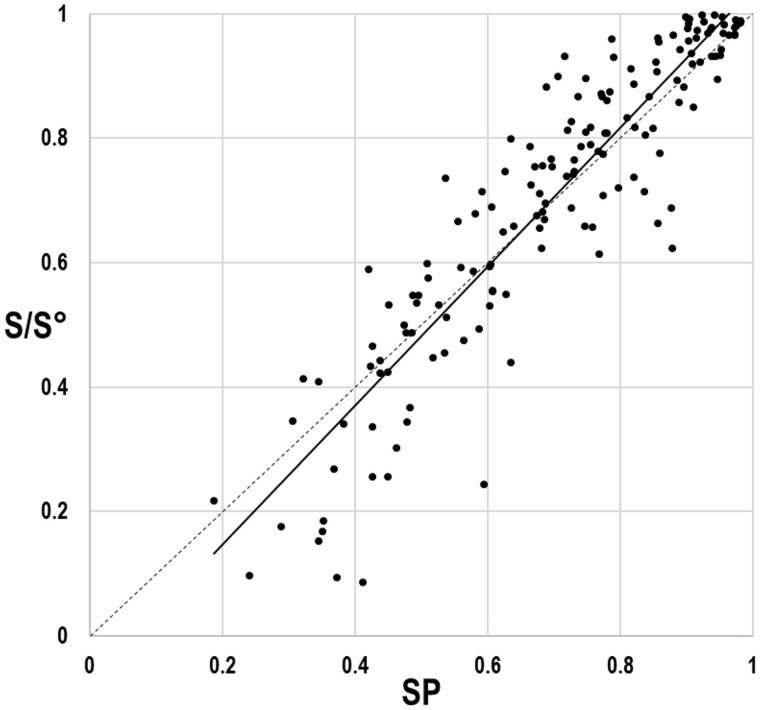
*S*/*S*° as a function of SP-score for simulated gold standard versus realigned MSAs. The 160 data points represent 40 simulated (gold standard) MSAs, each of which is compared to four different realigned MSAs of the corresponding simulated sequences. The solid line corresponds to the regression line and the dotted line to *y* = *x.*

Using the procedure described in Section 2, we created 40 simulated gold standard MSAs, each with a single associated structure. We realigned the sequences of each MSA using four programs: GISMO (v3.1) (Neuwald and Altschul, 2016), Kalign 3 (Lassmann, 2020), MAFFT (v7.471) (Katoh and Standley, 2014) and MUSCLE (v3.7) (Edgar, 2004). To compute each realigned MSA’s distance from its associated gold standard, we calculated an SP-score (from ‘Sum of the Pairs’), which is the proportion of aligned pairs of residues within the gold standard that are aligned identically within the realigned MSA. We then used eCOMPASS to compare each realigned MSA to its corresponding gold standard MSA. As described above, given two MSAs eCOMPASS generates directly comparable scores, which we here denote as *S* for the realigned MSA and as *S°* for the gold standard MSA. Notably, as expected, in all cases the *S*-score is less than the *S°*-score. To study how well the relative values of *S* and *S*° correspond to the distance between the realigned and gold standard MSAs, we plot, in Figure 2, *S/S*° versus SP for each case. There is clearly a strong and close to linear correlation between *S*/*S*° and SP, with the Pearson correlation coefficient equal to 0.92. The regression line has a slope of 1.117 and a *y* intercept of −0.077, suggesting that *S*/*S*° is a good and relatively direct proxy for gold standard distance. Hence, for real protein sequence alignments, where we do not have gold standards for comparison, we may use comparable *S*-scores as proxies for alignment accuracy.

### 3.5 CDD versus JackHMMER MSA analyses

**Fig. 3.**
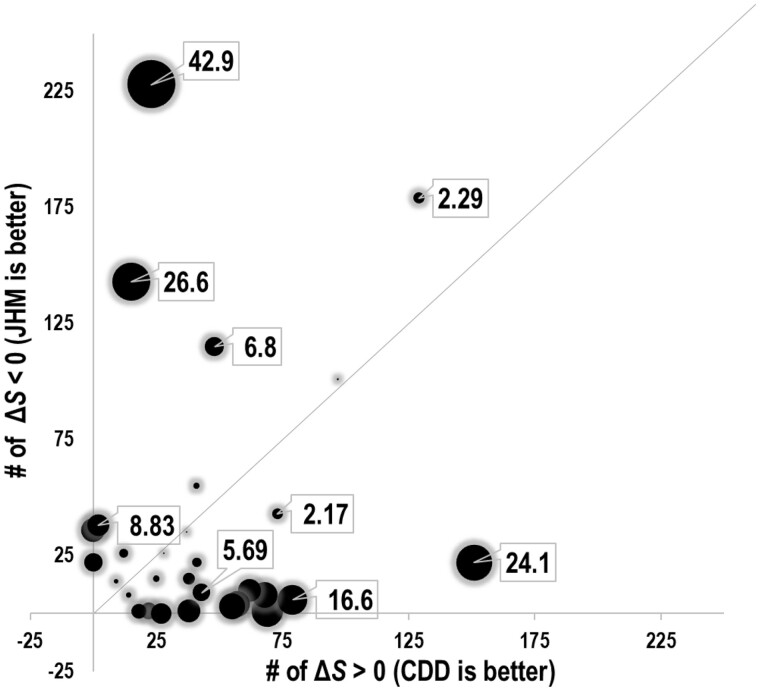
eCOMPASS analysis of CDD versus JackHMMER (JHM) MSAs. Data points represent 31 comparisons with the *x* and *y* axes corresponding to the numbers of reference structures for which Δ*S* > 0 and Δ*S* < 0, respectively. Hence, data points below and above the diagonal line correspond to analyses favoring the CDD and JHM MSA, respectively. The area of each bubble is proportional to −log_10_(*P*), the values of which are indicated for several data points

To further explore the utility and robustness of eCOMPASS, we compared the 40 CDD MSAs, upon which our simulated MSAs were based, to corresponding MSAs aligned with JackHMMER (JHM) (Johnson *et al.*, 2010) using an arbitrary sequence as the query (Supplementary Table S1). To reduce sequence redundancy, we removed from each MSA all but one sequence among those sharing ≥95% sequence identity using either cd-hit (Fu *et al.*, 2012) or PurgeMSA (Neuwald *et al.*, 2020). Note that this analysis allows the inclusion of more reference structures because, unlike the CDD versus Pfam analysis, the number of structures included was not predefined by Pfam. To identify domains for which a clearly significant distinction was at least possible, we focused on 31 of the 40 domains having at least 18 distinct structures, which could, in principle, yield a two-tailed binomial probability *P* < 10^−5^. Among these the CDD MSA was significantly better at the *P* < 10^−3^ level for 12 domains whereas the JHM MSA was significantly better for 6 domains (Fig. 3).

**Fig. 4.**
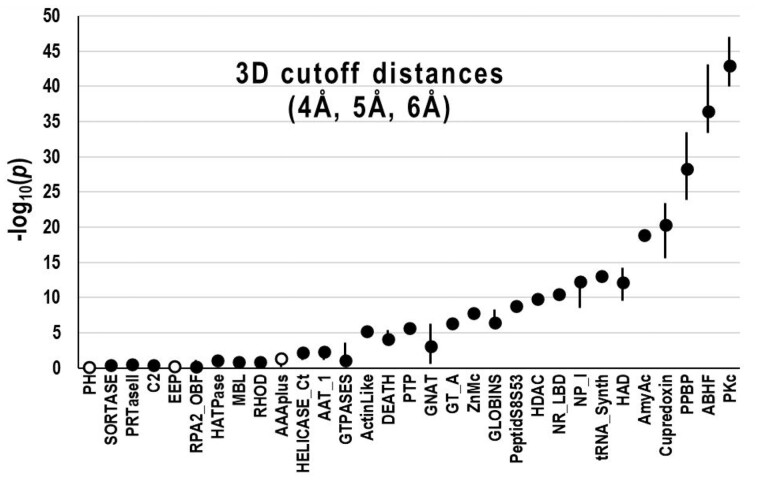
Influence of the 3D contact cutoff on eCOMPASS results. Plots indicate probabilities for CDD MSAs versus JHM MSAs using 4, 5 and 6 Å cutoffs. Circles correspond to median values and vertical lines to the high and low values. Closed or open circles indicate that the MSAs considered better are consistent or inconsistent, respectively, across the three settings. Domains are ordered left to right by the maximum of their three −log_10_(*P*) values.

**Fig. 5.**
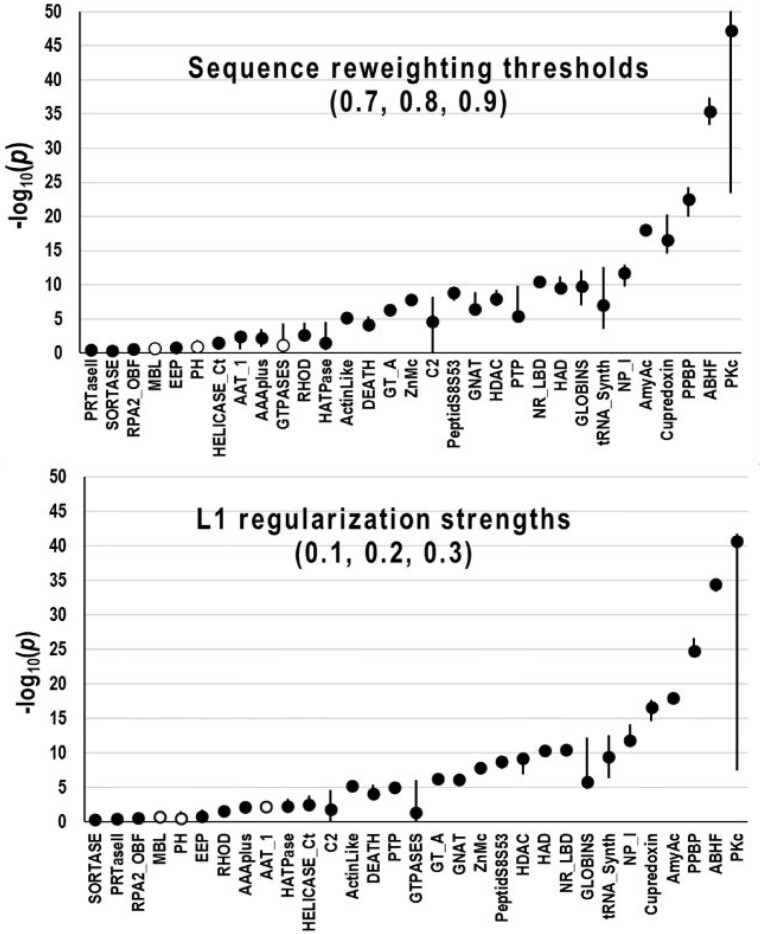
Influence of DCA hyperparameter settings on results. Plots indicate probabilities for CDD MSAs versus JHM MSAs using the three settings indicated. Circles correspond to median values and vertical lines to the high and low values. Closed or open circles indicate that the MSAs considered better are consistent or inconsistent, respectively, across the three settings. (**top**) CCMpred reweighting thresholds. (**bottom**) CCMpred L1 regularization strengths.

To evaluate the robustness of eCOMPASS, we reran each of these analyses using various CCMpred hyperparameter settings. (Another variable is the DCA implementation used, which, however, is too technically challenging to investigate here.) Using either flat (uniform) priors or Jeffreys uninformed priors [28] yielded essentially identical results (Supplementary Fig. S1). We also ran eCOMPASS with maximum residue pair 3D contact cutoffs of 4, 5 and 6 Å (Fig. 4), with alternative CCMpred sequence reweighting thresholds of 70%, 80% and 90% (Fig. 5, top), and with L1 regularization strengths of 0.1, 0.2 and 0.3 (Fig. 5, bottom). Notably, in only one case did two different parameter settings yield conflicting results both at a significance level ≤0.01. This arose for the L1 regularization parameter and the AAT_1 domain, for which conflicting results were reported with *P*-values of 0.005 and 0.002.

The observed variability in the binomial probability yielded by different parameter settings is likely due to changes in the implicit nature of the MSAs, of the ICA array or of both. For example, decreasing the CCMpred reweighting threshold (Seemayer *et al.*, 2014) is likely to decrease the DCA signal from highly populated subgroups.

## 4 Discussion

eCOMPASS computes a statistical score ($\bar{\Delta S}$) that compares the accuracy of two large MSAs and that is based on all the aligned sequences and on a set of reference structures. This score exploits the DC signal implicit in each alignment and whose strength presumably depends on the degree to which homologous residues are accurately aligned. eCOMPASS’s strategy constitutes a departure from current approaches. These typically rely upon a benchmark set, consisting of a small number of sequences aligned using structural data. However, they are essentially blind to the alignment accuracy of sequences absent from the set. Unlike other programs for assessing MSA quality (Ahola *et al.*, 2008; Lassmann and Sonnhammer, 2005; O'Sullivan *et al.*, 2003; Pei and Grishin, 2001; Song *et al.*, 2006; Thompson *et al.*, 2001), eCOMPASS provides measures of statistical significance, can handle extremely large MSAs, requires neither a gold standard MSA nor a structural alignment, and can assess the alignment quality of subgroups within an MSA.

Almost all multiple alignment construction methods employ some objective function of alignment quality which they attempt to optimize. For assessing the relative accuracy of two multiple alignments, relying upon the objective function used for either's construction will of course bias the results, so it is best to seek an independent measure. The congruence of structural contacts with alignment-derived DCA scores provides a convenient such measure, and one that avoids reliance upon a set of gold standard alignments.

Several recent multiple alignment construction methods (Muntoni *et al.*, 2020; Talibart and Coste, 2020a, 2020b; Wilburn and Eddy, 2020) incorporate DCA models into the objective functions they seek to optimize. To the extent that these models have been derived from particular structures, applying eCOMPASS to their evaluation using these very structures is likely to bias eCOMPASS's results in favor of the resulting multiple alignments. How to extend eCOMPASS to the comparison of such multiple alignments, or at least how to mitigate any confounding effects, is a question for further research. However, none of the alignments of real proteins studied here were constructed with the use of a Potts model.

Recently, Muntoni *et al.* (2020), in comparing the alignments constructed by their program DCAalign to those produced by other programs, used one method very similar in spirit to that of eCOMPASS. From alignment-derived pairwise coupling scores, they predicted contacting residue pairs and then, with reference to a known structure, plotted the true positive prediction rate as a function of the number of predictions made. It should be possible to derive from the resulting graphs a statistically based measure, similar to our Δ*S*, for the relative accuracy of the two alignments. Following, for example, the approach of Schäffer *et al*. (2001), one could calculate a ROC (receiver operating characteristic) score from a variant of each graph, and then infer *P*-values for the difference of these scores. Whether such a statistical approach is superior to the one taken here is an avenue for further study.

Ideally, eCOMPASS should be applied using a set of reference structures representing diverse subgroups within a superfamily, as in the examples here. Then, in addition to providing an assessment of overall alignment accuracy, eCOMPASS can identify those subgroups that are least accurately aligned, as an aid to improving MSA methods. This raises the issue of multiple conformations for the same protein, which is a major concern for DCA. A future version of eCOMPASS might provide the option of choosing the highest DC score among alternative conformations for each residue pair. In order to investigate directly coupled residue pairs corresponding to a subgroup-specific conformation, such as we reported recently (Tondnevis *et al.*, 2020), it may be useful to apply eCOMPASS to subgroup alignments within a superfamily MSA.

For MSA methods that fail to incorporate information from DCA into their objective functions, the statistical significance of the agreement between DC scores and 3D contacts within available structures serves as a measure of alignment accuracy that is independent of the criteria used in constructing the MSA. In any case, eCOMPASS should be uniquely useful for evaluating the extremely large MSAs typically required for deep learning protein sequence analyses and for statistical analyses requiring a vast amount of sequence data.

## Supplementary Material

btab374_Supplementary_Data
