# Supplementary material for: eCOMPASS: evaluative comparison of multiple protein alignments by statistical score
Source: Bioinformatics. 2021 May 13;37(20):3456–63. doi: 10.1093/bioinformatics/btab374 (PMC8545322; doi:10.1093/bioinformatics/btab374)

eCOMPASS: evaluative comparison of multiple protein alignments by statistical score

Andrew F. Neuwald[1,*] , Bryan D. Kolaczkowski[2] and Stephen F. Altschul[3]

[1]Department of Biochemistry & Molecular Biology, University of Maryland School of Medicine, Baltimore, MD 21201, USA, [2]Department of Microbiology & Cell Science, University of Florida, Gainesville, FL 32611, USA and [3]Computational Biology Branch, National Center for Biotechnology Information, National Library of Medicine, National Institutes of Health, Bethesda, Maryland, USA

Figure S1. Influence of priors on on results. Plots indicate probabilities for CDD MSAs versus JHM MSAs using flat (uniform) priors or Jeffreys uninformed priors.

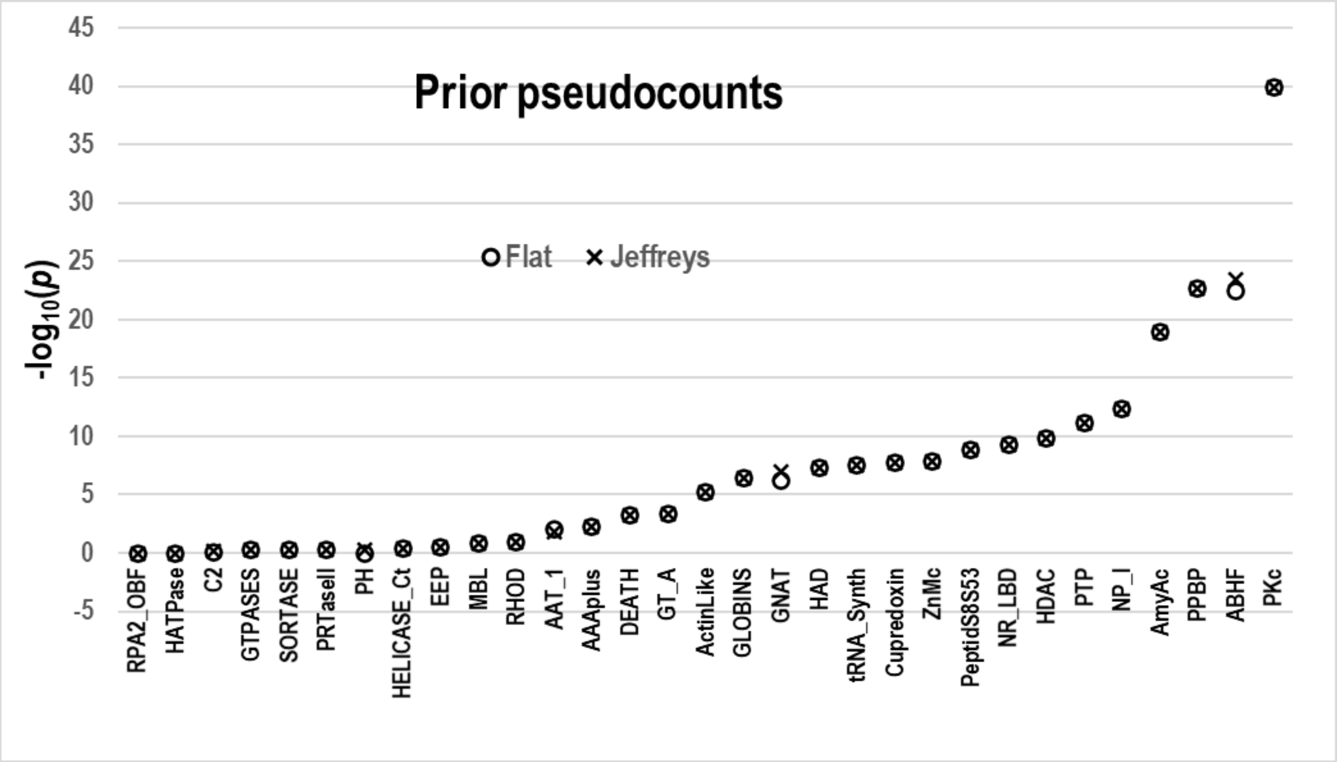

Supplement: btab374_Supplementary_Data [file btab374_supplementary_data.zip › neuwald_figS1.pdf]
