# Supplementary material for: eCOMPASS: evaluative comparison of multiple protein alignments by statistical score
Source: Bioinformatics. 2021 May 13;37(20):3456–63. doi: 10.1093/bioinformatics/btab374 (PMC8545322; doi:10.1093/bioinformatics/btab374)
Supplement: btab374_Supplementary_Data [file btab374_supplementary_data.zip › neuwald_suppl_data.pdf]

# eCOMPASS: evaluative comparison of multiple protein alignments by statistical score

Andrew F. Neuwald[1,*] , Bryan D. Kolaczkowski[2] and Stephen F. Altschul[3]

[1]Department of Biochemistry & Molecular Biology, University of Maryland School of Medicine, Baltimore, MD 21201, USA, [2]Department of Microbiology & Cell Science, University of Florida, Gainesville, FL 32611, USA and [3]Computational Biology Branch, National Center for Biotechnology Information, National Library of Medicine, National Institutes of Health, Bethesda, Maryland, USA

## Supplementary data: COMPASS output files

```
COMPASS: Comparison of multiple protein alignments by statistical score
     MSA 1(cddSFTS): 237 columns; MSA 2(pfSFTS): 309 columns; 35560 sequences.
========== Structural alignment percent identities ==========
                 MSA1     ---      MSA2     ---
n        pdbid   avg%ID   max%ID   avg%ID   max%ID
1        2W5Q_A  17.7     31.7     15.7     29.7
2        5WCX_A  13.1     17.2     11.0     16.1
3        4UOP_A  17.7     31.7     16.9     29.7
4        3LXQ_A  18.8     26.0     17.2     24.9
5        5FGN_A  16.3     41.5     14.0     36.7
6        6BNE_A  16.4     41.5     13.8     36.7
7        6A83_A  16.0     27.6     13.5     22.9
8        6S20_C  22.7     32.9     19.0     29.3
9        4MHX_A  22.1     29.9     18.3     24.1
10       4UPK_A  23.8     46.8     20.0     40.7
11       4UPH_A  25.0     46.8     20.2     40.7
12       4UPL_A  23.8     37.1     20.6     32.8
13       1FSU_A  21.7     32.9     19.6     29.3
14       6IOZ_A  24.8     47.1     21.1     37.5
15       6HHM_A  25.3     47.1     21.2     37.5
16       6HR5_A  23.1     32.1     19.4     26.8
17       5G2V_A  23.5     32.9     20.3     29.4
18       4UG4_A  24.3     35.3     20.0     28.9
19       5G2T_A  20.3     29.5     18.1     24.3
20       6S21_A  23.1     31.2     19.2     25.1
21       5I5F_A  16.2     22.9     13.9     18.4
   Overall %ID=19.3
========= Distance difference scores ========
n        dD1      sd1      pairs    dD2      sd2      pairs    pdbid
1        1.68     0.36     296922   2.09     0.81     330740   2w5qA
2        2.40     2.06     296813   2.81     3.06     330435   5wcxA
3        1.56     0.07     296962   1.96     0.38     330818   4uopA
4        1.71     0.43     296783   1.89     0.17     330644   3lxqA
5        1.53     0.02     296951   2.08     0.76     330790   5fgnA
6        1.57     0.10     296900   2.22     1.20     330791   6bneA
7        1.56     0.08     296816   2.17     1.04     330677   6a83A
8        1.47     -        296924   1.86     0.05     330917   6s20C
9        1.43     -        296865   1.62     -        330718   4mhxA
10       1.20     -        296956   1.57     -        330882   4upkA
11       1.22     -        296945   1.53     -        330930   4uphA
12       2.99     3.42     296983   1.58     -        330934   4uplA
13       1.30     -        296842   1.54     -        330878   1fsuA
14       1.27     -        296929   1.58     -        330962   6iozA
15       1.27     -        296931   1.74     -        330946   6hhmA
16       1.40     -        296959   1.63     -        330933   6hr5A
17       1.22     -        296976   1.50     -        330954   5g2vA
18       1.29     -        296942   1.93     0.30     330757   4ug4A
19       1.31     -        296976   1.74     -        330962   5g2tA
20       1.28     -        296958   1.64     -        330888   6s21A
21       1.36     -        246849   1.94     0.31     316940   5i5fA
avg      1.52     -        294532   1.84     -        330166 (with outliers)
avg      1.40     -        294283   1.80     -        330112 (no outliers)
         MSA 1: 177 cols; MSA 2: 186 cols
============ STARC S-scores (z=4.0; m=5) ==============
     MSA 1: cddSFTS (MGS_cd00016)
     MSA 2: pfSFTS (PF00884)
n        S1       X1       d1       S2       X2       d2       dS       pdbid    cols     D        L
1        282.6    960      251      285.4    707      235      -2.8     2w5qA    210      313      21008
2        210.2*   479      178      177.5*   744      182      32.7*    5wcxA    210      319      20980
3        301.0    1008     264      299.8    752      245      1.2      4uopA    212      320      21417
4        333.9    888      276      331.3    452      233      2.6      3lxqA    213      334      21578
5        305.7    809      258      258.3    903      242      47.5     5fgnA    213      343      21600
6        274.2    825      242      236.5    896      228      37.7     6bneA    210      330      21011
7        309.2    639      251      267.5    671      234      41.7     6a83A    210      357      20995
8        374.6    661      278      383.7    469      258      -9.0     6s20C    222      361      23504
9        319.0    630      240      330.7    422      221      -11.7    4mhxA    213      305      21385
10       376.5    465      250      342.5    653      257      34.0     4upkA    217      331      22406
11       389.0    628      279      354.7    581      259      34.3     4uphA    217      342      22207
12       278.5*   446      202      331.7    497      230      -53.2*   4uplA    202      284      19379
13       377.0    482      255      369.7    497      254      7.3      1fsuA    220      340      22875
14       383.6    660      280      366.6    381      237      17.0     6iozA    218      345      22430
15       382.7    629      278      383.4    580      273      -0.7     6hhmA    217      352      22431
16       355.1    569      254      348.4    744      269      6.8      6hr5A    213      326      21580
17       391.9    609      281      377.0    389      245      14.9     5g2vA    213      349      21577
18       359.9    570      258      315.9    639      246      44.1     4ug4A    211      325      21180
19       356.7    589      259      366.6    590      263      -9.9     5g2tA    217      337      22427
20       368.4    502      255      357.3    430      241      11.1     6s21A    209      329      20801
21       278.0    499      213      249.6    363      183      28.5     5i5fA    193      288      17635
avg      343.1    664.3    259.1    327.6    585.2    243.3    15.5 +/- 19.7 (2 outliers removed)
avg      333.7    645.1    252.5    320.7    588.6    239.8    13.0 +/- 24.4 (no outliers removed)
    * = outliers with avg. distance difference >= 2.00 SD above the mean...
             ...(SD recomputed after removing outliers iteratively).
 omit outliers (14 vs  5): 2-tail BP=0.064; -log10(BP)=1.2
 keep outliers (15 vs  6): 2-tail BP=0.078; -log10(BP)=1.11
        run time: 234 seconds (3.90 minutes)
```

```
 COMPASS: Comparison of multiple protein alignments by statistical score
     MSA 1(cddC2): 102 columns; MSA 2(pfamC2): 103 columns; 72249 sequences.
========== Structural alignment percent identities ==========
```

| | | MSA1 | --- | MSA2 | --- |
|---|---|---|---|---|---|
| n | pdbid | avg%ID | max%ID | avg%ID | max%ID |
| 1 | 4QJ3_B | 19.6 | 35.1 | 19.1 | 31.2 |
| 2 | 6PBC_A | 21.2 | 34.0 | 21.1 | 34.4 |
| 3 | 1QAS_A | 22.0 | 35.1 | 21.9 | 34.4 |
| 4 | 5H4Y_A | 27.3 | 49.5 | 26.9 | 48.5 |
| 5 | 1UGK_A | 23.6 | 44.1 | 25.0 | 42.2 |
| 6 | 1W15_A | 24.7 | 46.1 | 24.9 | 45.5 |
| 7 | 2D8K_A | 29.0 | 49.5 | 28.3 | 48.5 |
| 8 | 3N5A_A | 26.4 | 46.1 | 26.3 | 45.5 |
| 9 | 1WFM_A | 17.6 | 25.7 | 15.4 | 23.5 |
| 10 | 2ENP_A | 25.0 | 44.6 | 25.4 | 43.1 |
| 11 | 3L9B_A | 15.5 | 20.8 | 12.7 | 18.1 |
| 12 | 4IHB_A | 20.3 | 36.5 | 23.4 | 38.8 |
| 13 | 2FK9_A | 19.6 | 27.7 | 18.6 | 27.0 |
| 14 | 6KZ8_A | 17.7 | 25.0 | 15.3 | 21.8 |
| 15 | 2NQ3_A | 18.8 | 24.5 | 18.3 | 25.3 |
| 16 | 1A25_A | 24.1 | 37.3 | 23.7 | 37.0 |
| 17 | 5UE8_A | 25.5 | 35.1 | 24.6 | 32.7 |
| 18 | 2CJT_A | 16.8 | 24.0 | 15.3 | 23.0 |
| 19 | 2BWQ_A | 24.1 | 48.0 | 23.0 | 44.7 |
| 20 | 1RH8_A | 27.4 | 48.0 | 26.5 | 44.7 |
| 21 | 3NSJ_A | 19.8 | 28.6 | 18.9 | 26.6 |
| 22 | 2NSQ_A | 27.0 | 37.6 | 27.4 | 38.9 |
| 23 | 4MJJ_A | 26.0 | 35.6 | 27.4 | 35.3 |
| 24 | 4LDC_A | 27.6 | 44.1 | 27.7 | 42.7 |
| 25 | 5IZ5_A | 22.6 | 31.0 | 22.9 | 31.7 |
| 26 | 6IEJ_A | 21.4 | 31.0 | 21.9 | 32.0 |
| 27 | 4V29_A | 22.2 | 28.4 | 23.4 | 30.9 |
| 28 | 2EP6_A | 25.6 | 38.6 | 25.7 | 41.8 |
| 29 | 5YQ3_A | 21.6 | 28.4 | 22.3 | 31.0 |
| 30 | 2DMH_A | 20.0 | 36.5 | 22.6 | 38.8 |
| 31 | 3JZY_A | 23.4 | 38.6 | 23.5 | 41.8 |
| 32 | 2B3R_A | 22.2 | 37.6 | 21.5 | 33.0 |
| 33 | 3PYC_A | 18.6 | 26.5 | 17.3 | 25.8 |
| 34 | 6EI6_A | 15.2 | 23.5 | 17.1 | 24.5 |

Overall %ID=22.3

```
========= Distance difference scores ========
```

| n | dD1 | sd1 | pairs | dD2 | sd2 | pairs | pdbid |
|---|---|---|---|---|---|---|---|
| 1 | 1.32 | - | 46297 | 1.39 | - | 36536 | 4qj3B |
| 2 | 1.19 | - | 46393 | 1.47 | - | 36529 | 6pbcA |
| 3 | 1.04 | - | 46391 | 1.20 | - | 36526 | 1qasA |
| 4 | 1.07 | - | 46430 | 1.13 | - | 36562 | 5h4yA |
| 5 | 1.46 | 0.02 | 46430 | 1.52 | - | 36574 | 1ugkA |
| 6 | 1.17 | - | 46430 | 1.07 | - | 36575 | 1w15A |
| 7 | 1.19 | - | 46430 | 1.26 | - | 36562 | 2d8kA |
| 8 | 1.15 | - | 46430 | 1.02 | - | 36575 | 3n5aA |
| 9 | 1.58 | 0.13 | 46430 | 1.64 | 0.08 | 36575 | 1wfmA |
| 10 | 1.31 | - | 46430 | 1.37 | - | 36562 | 2enpA |
| 11 | 1.35 | - | 46310 | 1.30 | - | 36380 | 3l9bA |
| 12 | 1.13 | - | 46305 | 1.09 | - | 36376 | 4ihbA |
| 13 | 1.14 | - | 46127 | 1.27 | - | 36218 | 2fk9A |
| 14 | 1.44 | 0.01 | 46260 | 1.62 | 0.07 | 36343 | 6kz8A |
| 15 | 1.20 | - | 46061 | 1.22 | - | 36130 | 2nq3A |
| 16 | 1.03 | - | 46414 | 1.09 | - | 36559 | 1a25A |
| 17 | 1.07 | - | 46181 | 1.09 | - | 36250 | 5ue8A |
| 18 | 1.04 | - | 46053 | 1.08 | - | 36122 | 2cjtA |
| 19 | 1.01 | - | 46430 | 1.17 | - | 36587 | 2bwqA |
| 20 | 3.38 | 1.64 | 46430 | 4.63 | 2.31 | 36587 | 1rh8A |
| 21 | 1.26 | - | 46289 | 1.27 | - | 36360 | 3nsjA |
| 22 | 1.11 | - | 46306 | 1.11 | - | 36449 | 2nsqA |
| 23 | 1.04 | - | 46430 | 1.16 | - | 36587 | 4mjjA |
| 24 | 1.16 | - | 46430 | 1.08 | - | 36575 | 4ldcA |
| 25 | 1.02 | - | 46378 | 1.07 | - | 36508 | 5iz5A |
| 26 | 1.03 | - | 46378 | 1.12 | - | 36508 | 6iejA |
| 27 | 1.11 | - | 46181 | 1.11 | - | 36250 | 4v29A |
| 28 | 1.18 | - | 46181 | 1.21 | - | 36250 | 2ep6A |
| 29 | 7.76 | 5.32 | 46318 | 8.29 | 5.05 | 36381 | 5yq3A |
| 30 | 1.28 | - | 46305 | 1.22 | - | 36376 | 2dmhA |
| 31 | 1.04 | - | 46253 | 1.05 | - | 36322 | 3jzyA |
| 32 | 1.09 | - | 46430 | 1.18 | - | 36575 | 2b3rA |
| 33 | 1.14 | - | 46167 | 1.15 | - | 36278 | 3pycA |
| 34 | 1.24 | - | 46430 | 1.28 | - | 36575 | 6ei6A |
| avg | 1.43 | - | 46328 | 1.53 | - | 36445 | (with outliers) |
| avg | 1.12 | - | 46314 | 1.15 | - | 36426 | (no outliers) |

MSA 1: 57 cols; MSA 2: 51 cols

============= STARC S-scores (z=4.0; m=5) ===============

MSA 1: cddC2 (MGS_cd00030)

MSA 2: pfamC2 (PF00168)

| n | S1 | X1 | d1 | S2 | X2 | d2 | dS | pdbid | cols | D | L |
|---|---|---|---|---|---|---|---|---|---|---|---|
| 1 | 71.3* | 176 | 76 | 65.8* | 111 | 63 | 5.5* | 4qj3B | 81 | 104 | 2891 |
| 2 | 81.2 | 201 | 84 | 74.9* | 125 | 70 | 6.3* | 6pbcA | 88 | 114 | 3437 |
| 3 | 81.5 | 198 | 85 | 82.9 | 117 | 73 | -1.3 | 1qasA | 86 | 114 | 3274 |
| 4 | 107.8 | 251 | 104 | 119.1 | 144 | 94 | -11.3 | 5h4yA | 97 | 129 | 4212 |
| 5 | 89.0* | 244 | 93 | 98.5* | 130 | 82 | -9.5* | 1ugkA | 89 | 119 | 3525 |
| 6 | 88.8 | 173 | 85 | 97.5 | 112 | 79 | -8.7 | 1w15A | 81 | 107 | 2877 |
| 7 | 88.3 | 202 | 85 | 96.8 | 145 | 81 | -8.4 | 2d8kA | 97 | 117 | 4214 |
| 8 | 93.6 | 195 | 90 | 104.1 | 126 | 84 | -10.5 | 3n5aA | 87 | 113 | 3348 |
| 9 | 57.0* | 246 | 68 | 66.6* | 101 | 57 | -9.6* | 1wfmA | 82 | 89 | 2953 |
| 10 | 72.0* | 244 | 77 | 75.0* | 136 | 68 | -3.0* | 2enpA | 90 | 100 | 3611 |
| 11 | 56.4* | 164 | 59 | 68.1 | 87 | 55 | -11.7* | 3l9bA | 76 | 76 | 2502 |
| 12 | 54.6 | 137 | 57 | 66.3 | 81 | 54 | -11.6 | 4ihbA | 73 | 75 | 2295 |
| 13 | 84.7 | 278 | 92 | 89.4 | 160 | 81 | -4.7 | 2fk9A | 94 | 117 | 3943 |
| 14 | 56.4* | 124 | 56 | 66.2* | 88 | 55 | -9.8* | 6kz8A | 80 | 83 | 2780 |
| 15 | 82.2 | 275 | 94 | 97.5 | 121 | 81 | -15.2 | 2nq3A | 85 | 114 | 3160 |
| 16 | 79.9 | 198 | 79 | 86.8 | 100 | 68 | -7.0 | 1a25A | 83 | 96 | 3027 |
| 17 | 97.8 | 146 | 84 | 105.1 | 115 | 81 | -7.3 | 5ue8A | 88 | 111 | 3403 |
| 18 | 58.6 | 117 | 56 | 65.3 | 75 | 52 | -6.7 | 2cjtA | 74 | 75 | 2350 |
| 19 | 94.5 | 227 | 96 | 100.3 | 130 | 84 | -5.8 | 2bwqA | 89 | 121 | 3526 |
| 20 | 62.2* | 243 | 71 | 66.1* | 141 | 63 | -3.9* | 1rh8A | 96 | 104 | 4131 |
| 21 | 65.4 | 170 | 72 | 76.7 | 113 | 69 | -11.4 | 3nsjA | 79 | 101 | 2710 |
| 22 | 90.1 | 193 | 90 | 98.9 | 120 | 82 | -8.8 | 2nsqA | 83 | 113 | 3018 |
| 23 | 70.1 | 167 | 76 | 77.3 | 107 | 70 | -7.3 | 4mjjA | 74 | 101 | 2377 |
| 24 | 97.3 | 195 | 93 | 111.9 | 126 | 88 | -14.5 | 4ldcA | 87 | 117 | 3348 |
| 25 | 91.6 | 304 | 100 | 97.6 | 136 | 83 | -6.0 | 5iz5A | 91 | 120 | 3674 |
| 26 | 84.8 | 256 | 90 | 90.3 | 139 | 78 | -5.5 | 6iejA | 94 | 116 | 3942 |
| 27 | 92.4 | 197 | 91 | 106.4 | 121 | 85 | -14.0 | 4v29A | 85 | 115 | 3160 |
| 28 | 87.8 | 210 | 84 | 99.6 | 125 | 78 | -11.7 | 2ep6A | 92 | 106 | 3741 |
| 29 | 10.3* | 380 | 23 | 8.1* | 129 | 15 | 2.2* | 5yq3A | 96 | 46 | 4118 |
| 30 | 62.2 | 185 | 66 | 85.5 | 130 | 71 | -23.3 | 2dmhA | 92 | 98 | 3760 |
| 31 | 95.0 | 226 | 96 | 105.5 | 133 | 87 | -10.5 | 3jzyA | 93 | 129 | 3839 |
| 32 | 95.8 | 265 | 99 | 109.4 | 141 | 89 | -13.7 | 2b3rA | 96 | 129 | 4124 |
| 33 | 77.3 | 148 | 75 | 83.1 | 109 | 71 | -5.9 | 3pycA | 76 | 97 | 2509 |
| 34 | 61.5 | 203 | 71 | 85.2 | 129 | 74 | -23.7 | 6ei6A | 90 | 110 | 3618 |
| avg | 83.3 | 204.6 | 84.4 | 93.5 | 122.2 | 77.5 | -10.2 +/- 5.3 (9 outliers removed) | | | | |
| avg | 77.6 | 209.9 | 79.9 | 86.1 | 120.7 | 72.5 | -8.5 +/- 6.4 (no outliers removed) | | | | |

* = outliers with avg. distance difference >= 2.00 SD above the mean...

...(SD recomputed after removing outliers iteratively).

omit outliers ( 0 vs 25): 2-tail BP=6e-08; -log10(BP)=7.22

keep outliers ( 3 vs 31): 2-tail BP=7.7e-07; -log10(BP)=6.12

run time: 78 seconds (1.30 minutes)

COMPASS: Comparison of multiple protein alignments by statistical score
MSA 1(cddCuDXN): 110 columns; MSA 2(pfamCuDXN): 119 columns; 15418 sequences.

========== Structural alignment percent identities ==========

| | | MSA1 | --- | MSA2 | --- |
|---|---|---|---|---|---|
| n | pdbid | avg%ID | max%ID | avg%ID | max%ID |
| 1 | 4ENZ_A | 26.3 | 46.4 | 23.3 | 45.7 |
| 2 | 5N4L_A | 23.0 | 48.2 | 16.1 | 35.7 |
| 3 | 4BDV_A | 23.0 | 42.7 | 20.9 | 35.2 |
| 4 | 1SDD_B | 24.3 | 48.2 | 16.9 | 35.7 |
| 5 | 4BDV_A | 22.5 | 40.9 | 23.2 | 45.7 |
| 6 | 5TB7_A | 23.7 | 44.6 | 22.7 | 41.7 |
| 7 | 5ZL1_A | 24.6 | 46.0 | 23.4 | 41.2 |
| 8 | 4KNS_A | 25.3 | 49.0 | 23.3 | 48.1 |
| 9 | 3X1E_A | 23.7 | 49.0 | 22.1 | 48.1 |
| 10 | 3GDC_A | 26.6 | 34.3 | 26.8 | 37.2 |
| 11 | 1KV7_A | 22.7 | 37.9 | 21.2 | 35.7 |
| 12 | 2UXT_A | 20.2 | 34.0 | 18.8 | 35.7 |
| 13 | 5ZLL_A | 18.5 | 24.5 | 8.5 | 11.9 |
| 14 | 6KLG_A | 23.0 | 36.1 | 22.5 | 35.9 |
| 15 | 1ZPU_A | 26.3 | 45.4 | 25.6 | 44.0 |
| 16 | 3AW5_A | 23.3 | 35.0 | 22.3 | 34.3 |
| 17 | 5LWX_A | 25.9 | 39.8 | 24.6 | 42.1 |
| 18 | 3ZX1_A | 26.0 | 37.9 | 23.8 | 32.5 |
| 19 | 2XYB_A | 23.3 | 45.4 | 22.7 | 44.0 |
| 20 | 3G5W_A | 27.7 | 38.9 | 27.1 | 37.2 |
| 21 | 3T9W_A | 24.0 | 33.3 | 22.7 | 33.3 |

Overall %ID=22.9

========= Distance difference scores ========

| n | dD1 | sd1 | pairs | dD2 | sd2 | pairs | pdbid |
|---|---|---|---|---|---|---|---|
| 1 | 1.34 | 1.17 | 50350 | 0.85 | - | 22558 | 4enzA |
| 2 | 1.37 | 1.39 | 50350 | 0.87 | - | 22558 | 5n41A |
| 3 | 1.39 | 1.54 | 50350 | 0.85 | - | 22558 | 4bdvA |
| 4 | 1.35 | 1.21 | 50350 | 0.85 | - | 22558 | 1sddB |
| 5 | 1.42 | 1.77 | 50350 | 0.95 | - | 22558 | 4bdvA |
| 6 | 1.13 | - | 50312 | 1.02 | 0.38 | 22560 | 5tb7A |
| 7 | 1.07 | - | 50312 | 0.85 | - | 22560 | 5zl1A |
| 8 | 1.05 | - | 50312 | 0.91 | - | 22560 | 4knsA |
| 9 | 1.15 | - | 50303 | 0.95 | - | 22554 | 3x1eA |
| 10 | 1.02 | - | 50279 | 0.88 | - | 22534 | 3gdcA |
| 11 | 1.16 | - | 50327 | 0.94 | - | 22558 | 1kv7A |
| 12 | 1.10 | - | 50327 | 0.96 | 0.02 | 22558 | 2uxtA |
| 13 | 1.16 | - | 50170 | 0.85 | - | 22558 | 6klgA |
| 14 | 1.20 | 0.03 | 50266 | 0.94 | - | 22578 | 1zpuA |
| 15 | 1.30 | 0.86 | 50327 | 1.01 | 0.30 | 22558 | 3aw5A |
| 16 | 1.09 | - | 50218 | 0.85 | - | 22554 | 5lwxA |
| 17 | 1.12 | - | 50345 | 0.91 | - | 22558 | 3zx1A |
| 18 | 1.10 | - | 50275 | 1.46 | 2.99 | 22558 | 2xybA |
| 19 | 1.06 | - | 50279 | 0.84 | - | 22534 | 3g5wA |
| 20 | 1.27 | 0.65 | 50360 | 1.38 | 2.52 | 22558 | 3t9wA |
| avg | 1.19 | - | 50308 | 0.96 | - | 22556 | (with outliers) |
| avg | 1.19 | - | 50306 | 0.89 | - | 22556 | (no outliers) |

MSA 1: 77 cols; MSA 2: 53 cols

============= STARC S-scores (z=4.0; m=5) ===============

MSA 1: cddCuDXN (MGS_cd00920)
MSA 2: pfamCuDXN (PF07732)

| n | S1 | X1 | d1 | S2 | X2 | d2 | dS | pdbid | cols | D | L |
|---|---|---|---|---|---|---|---|---|---|---|---|
| 1 | 53.8 | 139 | 60 | 53.4 | 155 | 62 | 0.3 | 4enzA | 84 | 109 | 3123 |
| 2 | 31.8 | 54 | 31 | 31.0 | 41 | 28 | 0.8 | 5n41A | 63 | 57 | 1670 |
| 3 | 44.2 | 217 | 63 | 44.9 | 92 | 47 | -0.7 | 4bdvA | 88 | 113 | 3447 |
| 4 | 30.4 | 39 | 27 | 29.8 | 32 | 25 | 0.6 | 1sddB | 51 | 40 | 1035 |
| 5 | 49.0 | 206 | 61 | 44.3 | 79 | 44 | 4.7 | 4bdvA | 81 | 94 | 2894 |
| 6 | 67.3 | 192 | 72 | 71.7* | 89 | 59 | -4.4* | 5tb7A | 93 | 109 | 3869 |
| 7 | 62.2 | 111 | 61 | 60.5 | 72 | 52 | 1.8 | 5zl1A | 76 | 93 | 2513 |
| 8 | 69.3 | 140 | 66 | 67.2 | 80 | 55 | 2.1 | 4knsA | 86 | 99 | 3279 |
| 9 | 77.8 | 209 | 80 | 73.4 | 85 | 59 | 4.4 | 3x1eA | 91 | 108 | 3694 |
| 10 | 55.0 | 71 | 47 | 64.8 | 90 | 56 | -9.8 | 3gdcA | 90 | 106 | 3584 |
| 11 | 65.3 | 194 | 69 | 62.0 | 98 | 55 | 3.3 | 1kv7A | 93 | 103 | 3864 |
| 12 | 81.6 | 231 | 89 | 67.2 | 187 | 75 | 14.4 | 2uxtA | 97 | 132 | 4222 |
| 13 | 93.1 | 272 | 100 | 94.2 | 166 | 87 | -1.1 | 6klgA | 105 | 149 | 4969 |
| 14 | 80.7 | 222 | 83 | 71.8 | 89 | 58 | 8.9 | 1zpuA | 101 | 123 | 4589 |
| 15 | 63.4 | 194 | 72 | 56.9* | 79 | 51 | 6.5* | 3aw5A | 84 | 103 | 3102 |
| 16 | 83.6 | 180 | 80 | 81.6 | 95 | 65 | 2.0 | 5lwxA | 100 | 123 | 4489 |
| 17 | 81.0 | 251 | 86 | 73.3 | 201 | 77 | 7.8 | 3zx1A | 97 | 119 | 4226 |
| 18 | 81.7 | 205 | 81 | 80.8* | 88 | 62 | 0.9* | 2xybA | 98 | 116 | 4313 |
| 19 | 84.5 | 212 | 83 | 85.5 | 93 | 66 | -1.0 | 3g5wA | 99 | 121 | 4401 |
| 20 | 55.4 | 97 | 55 | 54.8* | 75 | 50 | 0.7* | 3t9wA | 82 | 106 | 2957 |
| avg | 65.2 | 171.8 | 67.9 | 62.8 | 103.4 | 56.9 | 2.4 +/- 5.3 (4 outliers removed) | | | | |
| avg | 65.6 | 171.8 | 68.3 | 63.4 | 99.3 | 56.6 | 2.1 +/- 5.1 (no outliers removed) | | | | |

* = outliers with avg. distance difference >= 2.00 SD above the mean...
...(SD recomputed after removing outliers iteratively).
omit outliers (12 vs 4): 2-tail BP=0.077; -log10(BP)=1.11
keep outliers (15 vs 5): 2-tail BP=0.041; -log10(BP)=1.38
run time: 39 seconds (0.65 minutes)

COMPASS: Comparison of multiple protein alignments by statistical score
MSA 1(cddHAD): 95 columns; MSA 2(pfHAD): 95 columns; 58031 sequences.

========== Structural alignment percent identities ==========

| | | MSA1 | --- | MSA2 | --- |
|---|---|---|---|---|---|
| n | pdbid | avg%ID | max%ID | avg%ID | max%ID |
| 1 | 3VAY_A | 23.5 | 36.7 | 22.5 | 35.8 |
| 2 | 2W43_A | 20.6 | 29.7 | 19.8 | 28.4 |
| 3 | 3UM9_A | 19.6 | 30.8 | 19.3 | 29.5 |
| 4 | 2B0C_A | 21.7 | 35.6 | 21.0 | 32.6 |
| 5 | 5AHX_A | 22.0 | 34.5 | 22.2 | 32.6 |
| 6 | 4DCC_A | 21.4 | 48.3 | 16.0 | 39.1 |
| 7 | 4JB3_A | 20.7 | 48.3 | 16.1 | 39.1 |
| 8 | 3NUQ_A | 18.0 | 23.9 | 19.2 | 25.3 |
| 9 | 3KZX_A | 24.7 | 31.9 | 23.3 | 30.5 |
| 10 | 3SD7_A | 19.2 | 28.0 | 18.6 | 27.4 |
| 11 | 4YGQ_A | 24.9 | 36.7 | 24.7 | 35.8 |
| 12 | 2GFH_A | 21.5 | 34.1 | 20.7 | 32.6 |
| 13 | 6W04_A | 20.5 | 31.9 | 21.2 | 31.6 |
| 14 | 4GIB_A | 19.6 | 25.3 | 19.6 | 26.3 |
| 15 | 4EEK_A | 20.3 | 31.9 | 21.0 | 31.6 |
| 16 | 2QLT_A | 19.3 | 33.3 | 19.3 | 29.5 |
| 17 | 3QU2_A | 20.2 | 33.3 | 20.4 | 28.4 |
| 18 | 2MSN_A | 18.9 | 28.0 | 18.5 | 27.4 |

Overall %ID=20.6

========= Distance difference scores ========

| n | dD1 | sd1 | pairs | dD2 | sd2 | pairs | pdbid |
|---|---|---|---|---|---|---|---|
| 1 | 1.12 | - | 42838 | 1.05 | - | 21905 | 3vayA |
| 2 | 1.40 | 0.18 | 42804 | 1.25 | - | 21785 | 2w43A |
| 3 | 1.15 | - | 42920 | 1.17 | - | 21897 | 3um9A |
| 4 | 1.27 | - | 42830 | 1.09 | - | 21897 | 2b0cA |
| 5 | 1.44 | 0.38 | 42877 | 1.22 | - | 21897 | 5ahxA |
| 6 | 1.47 | 0.52 | 42855 | 1.15 | - | 21869 | 4dccA |
| 7 | 1.35 | - | 42776 | 1.21 | - | 21869 | 4jb3A |
| 8 | 1.90 | 2.44 | 42897 | 2.33 | 3.02 | 21901 | 3nuqA |
| 9 | 1.12 | - | 42926 | 1.22 | - | 21854 | 3kzxA |
| 10 | 1.81 | 2.03 | 42900 | 1.87 | 1.62 | 21898 | 3sd7A |
| 11 | 1.07 | - | 42926 | 1.08 | - | 21905 | 4ygqA |
| 12 | 1.20 | - | 42905 | 1.17 | - | 21905 | 2gfhA |
| 13 | 1.34 | - | 42920 | 1.26 | - | 21903 | 6w04A |
| 14 | 1.23 | - | 42889 | 1.19 | - | 21897 | 4gibA |
| 15 | 1.24 | - | 42890 | 1.21 | - | 21904 | 4eekA |
| 16 | 1.34 | - | 42914 | 1.54 | 0.62 | 21865 | 2qltA |
| 17 | 1.37 | 0.05 | 42920 | 1.27 | - | 21902 | 3qu2A |
| 18 | 1.55 | 0.88 | 42905 | 1.72 | 1.17 | 21897 | 2msnA |
| avg | 1.35 | - | 42883 | 1.33 | - | 21886 | (with outliers) |
| avg | 1.27 | - | 42877 | 1.18 | - | 21885 | (no outliers) |

MSA 1: 75 cols; MSA 2: 55 cols

============= STARC S-scores (z=4.0; m=5) ===============

MSA 1: cddHAD (MGS_cd01427)
MSA 2: pfHAD (PF00702)

| n | S1 | X1 | d1 | S2 | X2 | d2 | dS | pdbid | cols | D | L |
|---|---|---|---|---|---|---|---|---|---|---|---|
| 1 | 75.1 | 167 | 65 | 77.5 | 125 | 62 | -2.4 | 3vayA | 83 | 73 | 3026 |
| 2 | 81.9 | 137 | 70 | 90.6 | 140 | 74 | -8.7 | 2w43A | 88 | 92 | 3412 |
| 3 | 94.4 | 199 | 84 | 98.3 | 145 | 79 | -3.9 | 3um9A | 90 | 97 | 3584 |
| 4 | 88.2 | 137 | 74 | 102.4 | 145 | 81 | -14.2 | 2b0cA | 85 | 93 | 3192 |
| 5 | 76.4 | 171 | 70 | 77.3 | 139 | 67 | -0.9 | 5ahxA | 77 | 79 | 2599 |
| 6 | 42.7 | 81 | 40 | 37.7 | 68 | 36 | 5.0 | 4dccA | 55 | 45 | 1243 |
| 7 | 40.8 | 80 | 40 | 35.4 | 76 | 37 | 5.3 | 4jb3A | 55 | 48 | 1239 |
| 8 | 51.0* | 158 | 53 | 42.6* | 244 | 55 | 8.4* | 3nuqA | 83 | 74 | 3060 |
| 9 | 94.2 | 138 | 75 | 91.5 | 139 | 74 | 2.7 | 3kzxA | 88 | 90 | 3423 |
| 10 | 39.7* | 98 | 40 | 77.6* | 131 | 64 | -38.0* | 3sd7A | 91 | 84 | 3669 |
| 11 | 86.4 | 211 | 80 | 91.8 | 138 | 74 | -5.5 | 4ygqA | 88 | 91 | 3426 |
| 12 | 90.8 | 179 | 78 | 79.4 | 129 | 67 | 11.4 | 2gfhA | 87 | 88 | 3346 |
| 13 | 67.2 | 137 | 61 | 76.0 | 133 | 64 | -8.8 | 6w04A | 88 | 86 | 3432 |
| 14 | 77.7 | 190 | 73 | 85.4 | 135 | 70 | -7.7 | 4gibA | 88 | 88 | 3417 |
| 15 | 68.6 | 180 | 66 | 78.2 | 139 | 66 | -9.6 | 4eekA | 85 | 81 | 3204 |
| 16 | 59.7 | 144 | 59 | 68.5* | 133 | 62 | -8.8* | 2qltA | 86 | 86 | 3260 |
| 17 | 55.7 | 143 | 56 | 79.2 | 123 | 65 | -23.5 | 3qu2A | 87 | 85 | 3354 |
| 18 | 77.5 | 139 | 66 | 73.2* | 140 | 64 | 4.3* | 2msnA | 89 | 85 | 3500 |
| avg | 74.3 | 153.6 | 66.6 | 78.6 | 126.7 | 65.4 | -4.3 +/- 8.9 (4 outliers removed) | | | | |
| avg | 70.4 | 149.4 | 63.9 | 75.7 | 134.6 | 64.5 | -5.3 +/- 11.9 (no outliers removed) | | | | |

* = outliers with avg. distance difference >= 2.00 SD above the mean...
...(SD recomputed after removing outliers iteratively).
omit outliers ( 4 vs 10): 2-tail BP=0.18; -log10(BP)=0.746
keep outliers ( 6 vs 12): 2-tail BP=0.24; -log10(BP)=0.624
run time: 49 seconds (0.82 minutes)

```
COMPASS: Comparison of multiple protein alignments by statistical score
     MSA 1(cddMBL): 188 columns; MSA 2(pfamMBL): 197 columns; 70293 sequences.
========== Structural alignment percent identities ==========
                MSA1     ---      MSA2     ---
n       pdbid   avg%ID   max%ID   avg%ID   max%ID
1       4EXS_A  14.1     40.7     11.6     30.4
2       6V5M_A  15.0     40.7     12.2     30.4
3       6CQS_A  14.3     26.7     10.7     19.3
4       6N36_A  16.7     34.4     12.8     34.8
5       5WCM_A  17.5     35.5     13.7     36.8
6       6MFI_A  18.2     48.6     13.6     36.8
7       6AUF_B  17.7     48.6     14.4     33.0
8       4D02_A  17.4     46.8     13.5     38.8
9       1E5D_A  16.4     42.8     12.5     41.6
10      6QRQ_A  14.6     22.0     11.2     18.9
11      1YCF_A  17.0     46.8     12.7     41.6
12      2CFU_A  14.4     46.5     11.0     44.6
13      4NUR_A  14.2     46.5     10.8     44.6
14      4V0H_A  15.5     20.4     8.4      29.4
15      5A0T_A  14.6     38.5     12.1     34.7
16      3ZQ4_A  14.5     38.5     12.9     34.7
17      4AD9_A  18.0     24.7     14.4     24.1
18      1QH3_A  17.7     39.0     8.7      27.3
19      1XM8_A  17.8     41.5     13.7     31.2
20      2QED_A  17.4     41.5     12.6     31.2
21      4EFZ_A  16.7     33.2     12.0     21.8
22      4YSB_A  17.7     48.8     8.9      27.3
23      5VE3_A  17.6     48.8     13.4     23.8
24      2ZO4_A  18.3     27.4     14.5     27.7
25      5HIO_A  16.5     24.6     13.0     23.0
26      6AO1_A  16.7     27.4     13.2     27.7
27      4KEP_A  15.9     31.7     12.1     18.6
28      6N9I_A  15.3     48.3     8.7      29.4
29      2R2D_A  15.1     48.3     11.3     23.9
30      2BIB_A  12.2     16.5     11.3     17.0
31      6I1D_A  11.6     24.4     10.0     15.7
32      3IE2_A  16.0     34.1     13.0     25.3
33      2XR1_A  14.6     34.1     12.3     25.3
34      2XF4_A  18.2     34.8     14.1     27.1
35      3X2Z_A  10.0     16.1     11.3     16.1
36      2ZWR_A  18.5     34.8     15.2     27.1
   Overall %ID=14.0
========= Distance difference scores ========
n       dD1     sd1     pairs   dD2     sd2     pairs   pdbid
1       1.98    -       123507  3.14    -       101261  4exsA
2       1.86    -       123399  4.01    0.20    101381  6v5mA
3       1.78    -       123594  3.38    -       101261  6cqsA
4       1.85    -       123595  2.86    -       101381  6n36A
5       1.78    -       123595  2.83    -       101381  5wcmA
6       1.73    -       123595  2.91    -       101381  6mfiA
7       1.86    -       123595  2.92    -       101381  6aufB
8       1.66    -       123589  3.15    -       101381  4d02A
9       1.65    -       123583  2.88    -       101381  1e5dA
10      1.85    -       123609  3.13    -       101381  6qrqA
11      1.70    -       123585  2.93    -       101381  1ycfA
12      1.70    -       123615  7.36    3.23    101381  2cfuA
13      1.69    -       123626  7.31    3.18    101381  4nurA
14      2.00    -       123500  3.82    0.03    101381  5a0tA
15      2.13    0.07    123475  3.95    0.14    101381  3zq4A
16      1.64    -       123550  3.69    -       101381  4ad9A
17      1.62    -       123493  3.27    -       101189  1xm8A
18      1.74    -       123283  3.93    0.13    100924  2qedA
19      1.97    -       123576  4.59    0.73    101381  4efzA
20      1.86    -       123572  3.52    -       101183  5ve3A
21      1.68    -       123525  3.08    -       101261  2zo4A
22      2.38    0.37    123445  3.01    -       101381  5hioA
23      6.17    4.72    123343  3.90    0.10    101381  6ao1A
24      1.77    -       123223  4.06    0.25    101381  4kepA
25      1.86    -       123264  4.36    0.52    101381  2r2dA
26      2.29    0.26    123206  5.18    1.25    101381  2bibA
27      2.08    0.02    123220  3.44    -       101381  6i1dA
28      2.15    0.11    123104  3.32    -       101381  3ie2A
29      2.15    0.10    122971  4.08    0.27    101381  2xr1A
30      1.68    -       123572  3.05    -       101304  2xf4A
31      4.09    2.33    123119  4.71    0.84    101304  3x2zA
32      1.70    -       118186  3.43    -       101304  2zwrA
avg     2.06    -       123285  3.79    -       101336 (with outliers)
avg     1.74    -       123230  3.28    -       101312 (no outliers)
        MSA 1: 93 cols; MSA 2: 85 cols
```

============= STARC S-scores (z=4.0; m=5) ===============

MSA 1: cddMBL (MGS_cd06262)

MSA 2: pfamMBL (PF00753)

| n | S1 | X1 | d1 | S2 | X2 | d2 | dS | pdbid | cols | D | L |
|---|---|---|---|---|---|---|---|---|---|---|---|
| 1 | 169.3* | 557 | 166 | 86.0 | 929 | 136 | 83.3* | 4exsA | 164 | 242 | 12598 |
| 2 | 138.1 | 376 | 138 | 64.0 | 435 | 96 | 74.0 | 6v5mA | 130 | 200 | 7793 |
| 3 | 169.0 | 620 | 172 | 61.9 | 779 | 110 | 107.1 | 6cqsA | 158 | 236 | 11669 |
| 4 | 125.9 | 485 | 139 | 71.7 | 681 | 117 | 54.2 | 6n36A | 121 | 179 | 6694 |
| 5 | 140.5 | 267 | 123 | 72.4 | 858 | 125 | 68.1 | 5wcmA | 128 | 182 | 7523 |
| 6 | 121.1 | 318 | 116 | 64.7 | 750 | 109 | 56.4 | 6mfiA | 124 | 163 | 7048 |
| 7 | 179.6 | 525 | 168 | 73.1 | 1092 | 130 | 106.6 | 6aufB | 176 | 261 | 14586 |
| 8 | 155.8 | 288 | 136 | 69.3 | 794 | 123 | 86.5 | 4d02A | 129 | 195 | 7656 |
| 9 | 149.2 | 275 | 132 | 69.7 | 796 | 125 | 79.5 | 1e5dA | 130 | 202 | 7775 |
| 10 | 172.7 | 527 | 159 | 88.1 | 838 | 127 | 84.6 | 6qrqA | 161 | 216 | 12144 |
| 11 | 167.6 | 187 | 122 | 71.8 | 919 | 125 | 95.8 | 1ycfA | 137 | 192 | 8671 |
| 12 | 79.1 | 483 | 98 | 31.9* | 790 | 74 | 47.2* | 2cfuA | 130 | 150 | 7828 |
| 13 | 87.8 | 495 | 104 | 36.7* | 507 | 67 | 51.2* | 4nurA | 131 | 150 | 7953 |
| 14 | 136.2* | 332 | 133 | 75.0 | 879 | 134 | 61.2* | 5a0tA | 129 | 200 | 7666 |
| 15 | 143.6* | 337 | 138 | 72.0 | 852 | 131 | 71.5* | 3zq4A | 131 | 209 | 7909 |
| 16 | 184.5 | 483 | 168 | 97.4 | 1040 | 151 | 87.1 | 4ad9A | 160 | 236 | 11973 |
| 17 | 149.5 | 383 | 140 | 92.7 | 788 | 135 | 56.8 | 1xm8A | 139 | 198 | 8938 |
| 18 | 172.1 | 447 | 164 | 74.6 | 898 | 132 | 97.6 | 2qedA | 147 | 235 | 10031 |
| 19 | 174.1* | 534 | 164 | 66.1* | 1557 | 140 | 108.0* | 4efzA | 163 | 233 | 12455 |
| 20 | 164.6 | 332 | 140 | 99.2 | 655 | 130 | 65.4 | 5ve3A | 144 | 202 | 9608 |
| 21 | 200.2 | 372 | 160 | 114.4 | 856 | 148 | 85.8 | 2zo4A | 171 | 240 | 13751 |
| 22 | 142.0* | 501 | 140 | 109.9 | 720 | 135 | 32.1* | 5hioA | 149 | 194 | 10374 |
| 23 | 118.5* | 282 | 106 | 81.8 | 600 | 107 | 36.6* | 6ao1A | 130 | 157 | 7824 |
| 24 | 167.7 | 375 | 149 | 72.1 | 977 | 128 | 95.7 | 4kepA | 166 | 251 | 12955 |
| 25 | 114.4 | 155 | 94 | 34.9* | 418 | 69 | 79.5* | 2r2dA | 118 | 168 | 6395 |
| 26 | 121.1* | 312 | 120 | 50.3* | 282 | 72 | 70.8* | 2bibA | 116 | 170 | 6182 |
| 27 | 117.8* | 417 | 125 | 36.4 | 723 | 86 | 81.5* | 6i1dA | 126 | 176 | 7308 |
| 28 | 149.6* | 404 | 145 | 62.4 | 1478 | 146 | 87.2* | 3ie2A | 144 | 221 | 9647 |
| 29 | 149.6* | 428 | 145 | 41.0 | 1068 | 106 | 108.6* | 2xr1A | 132 | 191 | 8057 |
| 30 | 196.9 | 482 | 175 | 110.0 | 1295 | 172 | 86.9 | 2xf4A | 177 | 269 | 14724 |
| 31 | 29.4* | 328 | 55 | 60.9* | 521 | 95 | -31.4* | 3x2zA | 137 | 193 | 8718 |
| 32 | 190.7 | 725 | 194 | 102.3 | 868 | 147 | 88.4 | 2zwrA | 173 | 269 | 14034 |
| avg | 163.7 | 414.8 | 149.7 | 81.6 | 851.1 | 129.4 | 82.0 +/- 16.4 (14 outliers removed) | | | | |
| avg | 146.2 | 407.2 | 138.4 | 72.3 | 832.6 | 119.6 | 73.9 +/- 27.9 (no outliers removed) | | | | |

* = outliers with avg. distance difference >= 2.00 SD above the mean...

...(SD recomputed after removing outliers iteratively).

omit outliers (18 vs 0): 2-tail BP=7.6e-06; -log10(BP)=5.12

keep outliers (31 vs 1): 2-tail BP=1.5e-08; -log10(BP)=7.81

run time: 252 seconds (4.20 minutes)

```
COMPASS: Comparison of multiple protein alignments by statistical score
     MSA 1(cddPH): 89 columns; MSA 2(pfamPH): 105 columns; 36099 sequences.
========== Structural alignment percent identities ==========
                MSA1     ---      MSA2     ---
n       pdbid   avg%ID   max%ID   avg%ID   max%ID
1       4H6Y_A  18.9     30.7     17.0     27.3
2       6NF1_A  17.0     25.0     14.5     20.6
3       6BNM_A  19.7     31.4     16.6     29.3
4       2PZ1_A  18.5     31.4     14.9     29.3
5       1RJ2_A  15.4     23.9     14.0     21.4
6       4F7H_A  13.5     21.6     12.1     17.6
7       4Y94_A  16.8     23.6     15.2     24.3
8       5HE0_A  17.2     25.0     15.6     24.5
9       2X18_A  17.8     27.3     16.4     25.3
10      2MDX_A  20.6     42.4     18.3     38.3
11      3FEH_A  17.4     26.4     14.9     21.4
12      4KAX_B  24.5     37.1     21.9     34.0
13      5A3F_A  19.2     28.4     16.6     25.7
14      1QQG_A  20.2     33.0     15.4     25.3
15      3TCA_A  16.6     25.0     15.3     22.5
16      1XDV_A  17.5     28.7     13.4     19.6
17      1FAO_A  22.8     41.4     20.7     37.9
18      4GZU_A  17.0     27.0     15.8     25.3
19      4H8S_A  18.8     27.0     16.2     26.3
20      1UPQ_A  21.6     31.8     19.1     28.3
21      4CKG_A  19.7     32.2     18.0     27.7
22      5C6R_A  17.2     23.3     15.9     21.6
23      3FEH_A  16.7     23.9     14.9     21.2
24      1WG7_A  22.5     33.0     20.0     29.9
25      1X1F_A  15.0     21.8     12.4     16.3
26      1EAZ_A  22.2     41.4     19.6     37.9
27      6FSF_A  16.7     24.7     15.8     26.7
28      2RSG_A  20.1     42.4     18.3     38.3
29      1XX0_A  17.0     25.0     21.3     34.3
30      3HW2_B  18.2     30.7     16.3     27.6
31      2LG1_A  15.4     23.9     13.7     22.3
   Overall %ID=17.4
========= Distance difference scores ========
n       dD1     sd1     pairs   dD2     sd2     pairs   pdbid
1       1.01    -       61369   1.27    -       43756   4h6yA
2       0.95    -       61481   1.68    0.25    43857   6nf1A
3       1.01    -       61481   2.64    2.77    43865   6bnmA
4       0.97    -       61481   1.71    0.31    43857   2pz1A
5       0.96    -       61481   1.23    -       43847   1rj2A
6       1.07    -       61502   1.34    -       43857   4f7hA
7       0.94    -       61413   1.23    -       43756   4y94A
8       1.02    -       61462   1.32    -       43736   5he0A
9       0.95    -       61518   1.25    -       43861   2x18A
10      1.32    0.84    61445   1.66    0.20    43819   2mdxA
11      1.00    -       61433   2.38    2.08    43847   3fehA
12      0.95    -       61502   1.81    0.59    43865   4kaxB
13      1.08    -       61502   1.36    -       43857   5a3fA
14      1.01    -       61359   1.56    -       43775   1qqgA
15      1.08    -       61502   1.64    0.15    43857   3tcaA
16      1.03    -       61361   1.44    -       43819   1xdvA
17      0.87    -       61413   1.20    -       43756   1faoA
18      1.44    1.34    61433   1.31    -       43756   4gzuA
19      1.04    -       61481   1.56    -       43731   4h8sA
20      0.89    -       61445   1.20    -       43819   1upqA
21      1.20    0.35    61398   1.61    0.08    43756   4ckgA
22      1.02    -       61369   1.35    -       43729   5c6rA
23      0.99    -       61527   2.25    1.74    43873   3fehA
24      1.03    -       61519   1.55    -       43838   1wg7A
25      1.29    0.71    61413   2.10    1.35    43847   1x1fA
26      0.89    -       61413   1.21    -       43756   1eazA
27      1.74    2.58    61481   1.21    -       43687   6fsfA
28      1.72    2.47    61369   1.90    0.83    43756   2rsgA
29      1.52    1.67    61474   1.39    -       43835   1xx0A
30      0.99    -       61507   1.66    0.20    43819   3hw2B
31      1.59    1.95    61502   2.14    1.45    43857   2lg1A
avg     1.12    -       61453   1.59    -       43808  (with outliers)
avg     0.99    -       61454   1.43    -       43807  (no outliers)
        MSA 1: 68 cols; MSA 2: 58 cols
```

============= STARC S-scores (z=4.0; m=5) ===============

MSA 1: cddPH (MGS_cd00900)

MSA 2: pfamPH (PF00169)

| n | S1 | X1 | d1 | S2 | X2 | d2 | dS | pdbid | cols | D | L |
|---|---|---|---|---|---|---|---|---|---|---|---|
| 1 | 57.1 | 170 | 63 | 52.0 | 192 | 62 | 5.1 | 4h6yA | 78 | 85 | 2642 |
| 2 | 65.5 | 169 | 72 | 43.1 | 102 | 49 | 22.4 | 6nf1A | 85 | 114 | 3186 |
| 3 | 56.3 | 180 | 65 | 24.4* | 201 | 45 | 32.0* | 6bnmA | 79 | 96 | 2740 |
| 4 | 64.0 | 202 | 74 | 41.4 | 101 | 47 | 22.6 | 2pz1A | 83 | 106 | 3043 |
| 5 | 68.8 | 192 | 77 | 63.3 | 117 | 63 | 5.5 | 1rj2A | 81 | 108 | 2879 |
| 6 | 49.8 | 150 | 58 | 43.8 | 92 | 47 | 6.0 | 4f7hA | 75 | 89 | 2441 |
| 7 | 65.9 | 198 | 78 | 58.7 | 237 | 77 | 7.2 | 4y94A | 80 | 112 | 2822 |
| 8 | 71.1 | 157 | 73 | 63.9 | 291 | 85 | 7.3 | 5he0A | 86 | 116 | 3245 |
| 9 | 83.1 | 93 | 69 | 73.6 | 128 | 72 | 9.5 | 2x18A | 85 | 120 | 3194 |
| 10 | 58.1* | 93 | 55 | 52.1 | 220 | 69 | 6.0* | 2mdxA | 85 | 109 | 3172 |
| 11 | 55.2 | 82 | 51 | 40.0* | 81 | 42 | 15.2* | 3fehA | 78 | 96 | 2668 |
| 12 | 72.3 | 149 | 74 | 71.5 | 86 | 61 | 0.8 | 4kaxB | 84 | 114 | 3101 |
| 13 | 63.6 | 209 | 76 | 58.7 | 236 | 76 | 4.9 | 5a3fA | 84 | 111 | 3114 |
| 14 | 47.9 | 135 | 56 | 36.4 | 82 | 41 | 11.5 | 1qqgA | 72 | 87 | 2241 |
| 15 | 57.1 | 91 | 53 | 53.6 | 229 | 68 | 3.5 | 3tcaA | 84 | 99 | 3130 |
| 16 | 35.9 | 140 | 49 | 34.9 | 81 | 40 | 0.9 | 1xdvA | 72 | 85 | 2225 |
| 17 | 78.8 | 163 | 80 | 67.9 | 122 | 67 | 10.9 | 1faoA | 87 | 122 | 3326 |
| 18 | 59.7* | 126 | 60 | 56.4 | 98 | 54 | 3.3* | 4gzuA | 76 | 85 | 2502 |
| 19 | 54.3 | 206 | 67 | 33.2 | 124 | 44 | 21.1 | 4h8sA | 86 | 103 | 3259 |
| 20 | 81.1 | 78 | 63 | 73.6 | 124 | 70 | 7.6 | 1upqA | 87 | 117 | 3343 |
| 21 | 64.9* | 149 | 67 | 57.5 | 256 | 74 | 7.4* | 4ckgA | 86 | 107 | 3250 |
| 22 | 71.5 | 119 | 67 | 66.2 | 122 | 65 | 5.3 | 5c6rA | 84 | 108 | 3086 |
| 23 | 69.6 | 213 | 78 | 48.3* | 57 | 42 | 21.3* | 3fehA | 86 | 109 | 3273 |
| 24 | 72.2 | 195 | 82 | 63.0 | 116 | 65 | 9.1 | 1wg7A | 82 | 117 | 2965 |
| 25 | 62.9* | 147 | 63 | 44.6* | 85 | 44 | 18.2* | 1x1fA | 84 | 98 | 3091 |
| 26 | 79.8 | 159 | 79 | 73.0 | 255 | 87 | 6.8 | 1eazA | 86 | 117 | 3259 |
| 27 | 57.2* | 152 | 64 | 54.9 | 186 | 67 | 2.3* | 6fsfA | 73 | 91 | 2310 |
| 28 | 44.6* | 83 | 45 | 48.6* | 118 | 53 | -4.0* | 2rsgA | 84 | 101 | 3093 |
| 29 | 28.6* | 152 | 48 | 69.8 | 120 | 70 | -41.2* | 1xx0A | 83 | 124 | 3038 |
| 30 | 73.0 | 90 | 64 | 63.0 | 90 | 59 | 10.0 | 3hw2B | 85 | 126 | 3189 |
| 31 | 62.2* | 184 | 76 | 10.7* | 51 | 20 | 51.5* | 2lg1A | 79 | 118 | 2739 |
| avg | 65.6 | 153.2 | 68.7 | 56.7 | 146.3 | 62.2 | 8.9 +/- 6.4 (11 outliers removed) | | | | |
| avg | 62.3 | 149.2 | 66.0 | 53.0 | 141.9 | 58.9 | 9.4 +/- 14.3 (no outliers removed) | | | | |

* = outliers with avg. distance difference >= 2.00 SD above the mean...

...(SD recomputed after removing outliers iteratively).

omit outliers (20 vs 0): 2-tail BP=1.9e-06; -log10(BP)=5.72

keep outliers (29 vs 2): 2-tail BP=4.6e-07; -log10(BP)=6.33

run time: 64 seconds (1.07 minutes)

```
COMPASS: Comparison of multiple protein alignments by statistical score
     MSA 1(cddPTS): 84 columns; MSA 2(pfamPTS): 90 columns; 9395 sequences.
========== Structural alignment percent identities ==========
                MSA1     ---      MSA2     ---
n       pdbid   avg%ID   max%ID   avg%ID   max%ID
1       3CZC_A  14.4     18.1     13.5     21.6
2       2WY2_D  16.6     42.2     17.0     41.4
3       2L2Q_A  14.8     31.3     14.1     29.9
4       4MGE_A  17.5     42.2     18.3     41.4
5       3NBM_A  14.5     21.4     16.4     20.2
6       1TVM_A  16.4     36.1     14.3     28.6
7       5GQS_A  17.5     36.1     16.7     28.6
8       1VKR_A  14.0     20.2     13.9     21.6
9       5DLE_A  22.4     47.6     23.4     48.8
10      2R48_A  22.4     46.4     21.2     44.3
11      4TN5_A  19.2     35.7     19.7     36.5
12      2KYR_A  22.1     42.9     21.7     44.2
13      2M1Z_A  23.4     47.6     23.0     48.8
   Overall %ID=18.0
========= Distance difference scores ========
n       dD1     sd1     pairs   dD2     sd2     pairs   pdbid
1       2.78    2.70    22123   2.94    0.29    14949   3czcA
2       2.10    -       22189   2.60    -       14971   2wy2D
3       2.42    0.91    22189   3.00    0.67    14973   2l2qA
4       2.06    -       22189   2.48    -       14973   4mgeA
5       2.24    0.01    22199   2.87    -       15000   3nbmA
6       2.34    0.52    22181   2.92    0.19    14832   1tvmA
7       2.23    -       22170   2.92    0.20    14909   5gqsA
8       2.12    -       22077   3.07    1.06    14859   1vkrA
9       2.08    -       22173   2.95    0.38    15013   5dleA
10      2.11    -       22173   2.91    0.10    15013   2r48A
11      2.12    -       22173   2.90    0.08    15020   4tn5A
12      2.37    0.63    22173   3.07    1.09    15020   2kyrA
13      2.10    -       22173   2.92    0.21    15020   2m1zA
avg     2.24    -       22168   2.89    -       14966 (with outliers)
avg     2.19    -       22172   2.89    -       14967 (no outliers)
        MSA 1: 65 cols; MSA 2: 54 cols
============= STARC S-scores (z=4.0; m=5) ===============
     MSA 1: cddPTS (MGS_cd00133)
     MSA 2: pfamPTS (PF02302)
n       S1      X1      d1      S2      X2      d2      dS      pdbid   cols    D       L
1       29.1*   288     57      41.0    253     63      -12.0*  3czcA   82      100     2944
2       46.9    143     54      34.1    259     56      12.9    2wy2D   77      85      2583
3       20.5    251     33      14.6    77      19      5.9     2l2qA   65      39      1801
4       50.9    126     56      38.2    268     62      12.7    4mgeA   78      93      2659
5       53.5    134     58      30.0    249     54      23.5    3nbmA   76      88      2525
6       28.8    84      32      18.6    153     31      10.2    1tvmA   74      60      2367
7       30.6    124     40      14.8    207     34      15.8    5gqsA   78      79      2647
8       28.1    219     45      11.4    46      17      16.7    1vkrA   71      64      2164
9       31.6    284     60      24.2    262     52      7.3     5dleA   77      97      2590
10      32.1    284     58      22.0    383     55      10.1    2r48A   77      93      2590
11      23.7    262     49      16.2    555     58      7.5     4tn5A   75      86      2453
12      22.4    260     48      20.1    223     43      2.4     2kyrA   77      90      2595
13      31.1    286     57      22.2    105     34      9.0     2m1zA   77      87      2594
avg     33.4    204.8   49.2    22.2    232.2   42.9    11.2 +/- 5.6 (1 outliers removed)
avg     33.0    211.2   49.8    23.6    233.8   44.5    9.4 +/- 8.4 (no outliers removed)
    * = outliers with avg. distance difference >= 2.00 SD above the mean...
            ...(SD recomputed after removing outliers iteratively).
 omit outliers (12 vs  0): 2-tail BP=0.00049; -log10(BP)=3.31
 keep outliers (12 vs  1): 2-tail BP=0.0034; -log10(BP)=2.47
        run time: 7 seconds (0.12 minutes)
```

```
COMPASS: Comparison of multiple protein alignments by statistical score
     MSA 1(cddRHOD): 89 columns; MSA 2(pfamRHOD): 107 columns; 61053 sequences.
========== Structural alignment percent identities ==========
                MSA1     ---      MSA2     ---
n       pdbid   avg%ID   max%ID   avg%ID   max%ID
1       2J6P_A  18.6     28.1     17.4     26.8
2       1C25_A  19.6     27.3     16.8     26.8
3       1T3K_A  17.3     28.4     15.3     22.4
4       3FS5_A  18.4     28.4     17.0     24.5
5       1GN0_A  20.3     31.0     18.1     29.3
6       1DP2_A  19.4     48.3     17.3     41.0
7       3UTN_X  15.2     33.7     15.0     33.3
8       3IPO_A  18.5     34.8     15.2     25.5
9       1URH_A  23.1     48.3     19.9     41.0
10      4JGT_A  18.6     31.5     16.8     27.6
11      3HZU_A  23.3     36.8     20.5     32.7
12      3AAX_A  17.7     31.5     16.0     26.4
13      2EG4_A  21.4     36.8     19.7     32.7
14      2VSW_A  19.2     34.1     17.5     31.1
15      2OUC_A  17.7     34.1     16.2     31.1
16      2HHG_A  21.9     36.0     19.2     25.3
17      6MXV_A  21.6     32.6     19.8     31.2
18      1QXN_A  21.8     36.0     19.5     31.6
19      4F67_A  18.2     28.1     16.4     24.0
20      3D1P_A  20.9     30.3     17.7     28.6
21      6BEV_A  22.2     33.7     19.5     29.1
22      2K0Z_A  17.3     23.8     18.1     25.6
23      3G5J_A  22.2     31.0     19.1     24.3
24      2KL3_A  20.7     31.4     20.3     32.2
25      3I3U_A  18.1     31.4     17.6     32.2
26      3NHV_A  21.3     29.5     18.2     25.6
27      2FSX_A  17.9     27.0     16.1     22.9
28      4OCG_A  20.5     33.3     19.5     31.1
29      3TP9_A  22.8     33.7     21.7     31.1
30      2JTQ_A  18.5     26.2     18.4     25.3
31      3I2V_A  19.3     30.3     17.8     26.2
32      2MOI_A  19.8     26.1     17.5     21.9
33      3IWH_A  21.3     32.6     19.3     31.2
   Overall %ID=18.9
========= Distance difference scores ========
n       dD1     sd1     pairs   dD2     sd2     pairs   pdbid
1       1.54    -       19180   2.62    0.69    18331   2j6pA
2       1.49    -       19180   2.69    0.84    18339   1c25A
3       2.28    1.04    19180   2.42    0.24    18345   1t3kA
4       1.46    -       19180   2.13    -       18339   3fs5A
5       1.68    -       19168   1.98    -       17977   1gn0A
6       1.61    -       19168   2.54    0.51    18323   1dp2A
7       1.72    0.02    19168   2.16    -       18314   3utnX
8       1.57    -       19168   2.06    -       18306   3ipoA
9       1.69    -       19168   2.22    -       18314   1urhA
10      1.60    -       19168   2.06    -       18306   4jgtA
11      1.52    -       19168   2.01    -       18316   3hzuA
12      1.56    -       19168   2.05    -       18314   3aaxA
13      1.39    -       19110   2.15    -       18215   2eg4A
14      2.17    0.83    19122   3.61    2.91    18346   2vswA
15      2.13    0.76    19123   3.73    3.16    18302   2oucA
16      1.29    -       19168   2.33    0.03    18275   2hhgA
17      1.30    -       19168   1.84    -       18306   6mxvA
18      1.87    0.30    19168   2.42    0.24    18051   1qxnA
19      2.04    0.60    19168   1.90    -       18306   4f67A
20      1.46    -       19168   2.31    -       18306   3d1pA
21      1.34    -       19168   2.62    0.69    18306   6bevA
22      1.93    0.40    19017   2.23    -       18215   2k0zA
23      2.04    0.60    19122   2.04    -       18243   3g5jA
24      1.79    0.14    19124   2.01    -       18171   2kl3A
25      1.49    -       19062   2.37    0.13    18145   3i3uA
26      1.45    -       19122   2.24    -       18115   3nhvA
27      4.32    4.74    19169   3.06    1.68    18306   2fsxA
28      1.27    -       19122   1.92    -       18100   4ocgA
29      1.27    -       19124   1.91    -       18171   3tp9A
30      1.59    -       19122   2.07    -       18140   2jtqA
31      1.37    -       19180   2.16    -       18333   3i2vA
32      1.97    0.48    19170   2.62    0.67    18256   2moiA
33      1.15    -       17035   1.92    -       18100   3iwhA
avg     1.71    -       19085   2.32    -       18249 (with outliers)
avg     1.57    -       19072   2.18    -       18236 (no outliers)
        MSA 1: 38 cols; MSA 2: 37 cols
```

============= STARC S-scores (z=4.0; m=5) ===============

MSA 1: cddRHOD (MGS_cd00158)

MSA 2: pfamRHOD (PF00581)

| n | S1 | X1 | d1 | S2 | X2 | d2 | dS | pdbid | cols | D | L |
|---|---|---|---|---|---|---|---|---|---|---|---|
| 1 | 55.8 | 87 | 49 | 45.7 | 111 | 48 | 10.1 | 2j6pA | 78 | 79 | 2698 |
| 2 | 51.9 | 147 | 59 | 50.3* | 132 | 56 | 1.6* | 1c25A | 74 | 86 | 2385 |
| 3 | 30.6* | 361 | 54 | 20.4 | 206 | 37 | 10.1* | 1t3kA | 75 | 69 | 2469 |
| 4 | 63.4 | 94 | 54 | 54.4 | 111 | 53 | 9.0 | 3fs5A | 81 | 88 | 2848 |
| 5 | 58.6 | 153 | 61 | 52.6 | 126 | 55 | 6.1 | 1gn0A | 85 | 94 | 3170 |
| 6 | 51.2 | 141 | 54 | 53.0 | 151 | 56 | -1.8 | 1dp2A | 76 | 76 | 2570 |
| 7 | 41.0 | 109 | 49 | 53.3 | 98 | 54 | -12.3 | 3utnX | 63 | 76 | 1710 |
| 8 | 48.7 | 114 | 47 | 42.3 | 51 | 35 | 6.4 | 3ipoA | 68 | 60 | 1975 |
| 9 | 69.5 | 94 | 60 | 72.1 | 155 | 71 | -2.6 | 1urhA | 82 | 100 | 2979 |
| 10 | 65.5 | 184 | 69 | 58.8 | 139 | 60 | 6.7 | 4jgtA | 87 | 97 | 3275 |
| 11 | 79.3 | 151 | 78 | 69.7 | 133 | 70 | 9.7 | 3hzuA | 81 | 109 | 2893 |
| 12 | 69.9 | 145 | 69 | 65.6 | 155 | 68 | 4.2 | 3aaxA | 85 | 107 | 3200 |
| 13 | 68.8 | 144 | 72 | 63.8 | 97 | 61 | 5.0 | 2eg4A | 73 | 97 | 2307 |
| 14 | 51.0* | 101 | 48 | 49.4* | 120 | 51 | 1.6* | 2vswA | 76 | 77 | 2556 |
| 15 | 51.8* | 74 | 45 | 44.0* | 91 | 44 | 7.8* | 2oucA | 63 | 60 | 1709 |
| 16 | 67.1 | 100 | 61 | 49.9 | 72 | 46 | 17.2 | 2hhgA | 81 | 106 | 2872 |
| 17 | 64.5 | 85 | 56 | 47.6 | 106 | 52 | 16.9 | 6mxvA | 77 | 98 | 2563 |
| 18 | 52.2 | 119 | 54 | 52.9 | 214 | 66 | -0.7 | 1qxnA | 87 | 102 | 3347 |
| 19 | 72.1 | 121 | 68 | 77.5 | 84 | 62 | -5.3 | 4f67A | 89 | 116 | 3500 |
| 20 | 82.5 | 109 | 72 | 68.0 | 154 | 73 | 14.5 | 3d1pA | 84 | 117 | 3111 |
| 21 | 88.8 | 176 | 85 | 71.1 | 108 | 64 | 17.7 | 6bevA | 88 | 117 | 3428 |
| 22 | 38.4 | 83 | 44 | 44.6 | 71 | 44 | -6.2 | 2k0zA | 76 | 101 | 2485 |
| 23 | 60.1 | 78 | 53 | 64.0 | 82 | 56 | -3.8 | 3g5jA | 73 | 89 | 2302 |
| 24 | 43.6 | 172 | 58 | 48.1 | 70 | 45 | -4.5 | 2k13A | 78 | 96 | 2652 |
| 25 | 47.7 | 83 | 44 | 17.8 | 84 | 28 | 29.9 | 3i3uA | 59 | 56 | 1440 |
| 26 | 69.8 | 141 | 70 | 52.8 | 77 | 50 | 17.0 | 3nhvA | 76 | 96 | 2503 |
| 27 | 50.7* | 119 | 54 | 44.9* | 68 | 42 | 5.7* | 2fsxA | 75 | 84 | 2461 |
| 28 | 74.2 | 146 | 74 | 63.6 | 103 | 61 | 10.6 | 4ocgA | 80 | 108 | 2775 |
| 29 | 87.7 | 91 | 71 | 66.4 | 85 | 60 | 21.4 | 3tp9A | 77 | 106 | 2561 |
| 30 | 34.7 | 65 | 38 | 44.7 | 76 | 46 | -10.0 | 2jtqA | 68 | 83 | 1962 |
| 31 | 72.2 | 142 | 70 | 53.3 | 204 | 67 | 18.9 | 3i2vA | 78 | 94 | 2669 |
| 32 | 37.6 | 224 | 57 | 30.3 | 61 | 32 | 7.2 | 2moiA | 84 | 99 | 3100 |
| 33 | 69.2 | 108 | 63 | 67.0 | 116 | 63 | 2.1 | 3iwhA | 84 | 106 | 3093 |
| avg | 61.9 | 123.5 | 60.7 | 55.4 | 110.5 | 55.2 | 6.5 +/- 10.3 (5 outliers removed) | | | | |
| avg | 59.7 | 129.1 | 59.4 | 53.3 | 112.5 | 53.8 | 6.4 +/- 9.6 (no outliers removed) | | | | |

* = outliers with avg. distance difference >= 2.00 SD above the mean...
...(SD recomputed after removing outliers iteratively).

omit outliers (19 vs 9): 2-tail BP=0.087; -log10(BP)=1.06

keep outliers (24 vs 9): 2-tail BP=0.014; -log10(BP)=1.87

run time: 64 seconds (1.07 minutes)
