# Supplementary material for: eCOMPASS: evaluative comparison of multiple protein alignments by statistical score
Source: Bioinformatics. 2021 May 13;37(20):3456–63. doi: 10.1093/bioinformatics/btab374 (PMC8545322; doi:10.1093/bioinformatics/btab374)
Supplement: btab374_Supplementary_Data [file btab374_supplementary_data.zip › neuwald_tableS1.pdf]

eCOMPASS: evaluative comparison of multiple protein alignments by statistical score

Andrew F. Neuwald[1,*] , Bryan D. Kolaczkowski[2] and Stephen F. Altschul[3]

[1]Department of Biochemistry & Molecular Biology, University of Maryland School of Medicine, Baltimore, MD 21201, USA, [2]Department of Microbiology & Cell Science, University of Florida, Gainesville, FL 32611, USA and [3]Computational Biology Branch, National Center for Biotechnology Information, National Library of Medicine, National Institutes of Health, Bethesda, Maryland, USA

Table S1. Protein domain superfamily CDD MSAs used for simulated MSAs and for comparisons with corresponding JHM MSAs.

Symbol	Description	CDD id	number		Structures[b]			CBP
			seqs	Cols[a]	total	cdd	jhm	$\log_{10}(p)$
AAAplus	AAA+ NTPases	-	81,354	204	116	73	43	2.2
AAT_1	proton amino acid transporter 1	cd01494	47,551	171	308	129	179	2.3
ABHF	α,β-hydrolase fold	-	52,449	209	158	15	143	**26.6**
ActinLike	Sugar kinase/HSP70/actin superfamily	cd00012	160,944	185	23	22	1	**5.2**
AmyAc	Alpha amylase catalytic domain	cd00551	157,702	260	70	69	1	**18.9**
C2	C2 domain	cd00030	28,115	102	53	27	26	0.0
Cupredoxin	Cupredoxin blue copper proteins	cd00920	131,837	117	85	79	6	**16.6**
DEATH	Death domains	cd08304	16,259	77	19	18	1	**4.1**
EEP	exo/endonucleases/phosphatases	cd08372	178,249	234	40	25	15	0.8
GLOBINS	globins	cd01067	52,207	118	76	68	8	**12.2**
GNAT	GNAT acetyltransferases	pfam12746	84,566	153	163	48	115	**6.8**
GTPASES	P-loop GTPases	-	38,363	164	198	97	101	0.1
GT_A	Glycosyltransferase family A	cd00761	55,996	185	22	0	22	**6.3**
HAD	Haloacid Dehalogenase-like Hydrolases	cd01427	289,568	178	72	62	10	**9.6**
HATPase	Histidine kinase-like ATPase domain	cd00075	67,948	101	38	12	26	1.5
HDAC	arginase-like histone deacetylases	cd09987	96,602	249	28	28	0	**8.1**
HELICASE_Ct	DEAD-like helicase C-terminal domain	cd09300	24,714	103	54	38	15	2.7
MBL	metallo-hydrolase-like_MBL-fold	cd06262	99,078	176	96	41	55	0.7
NP_I	nucleoside phosphorylase-I domain	cd09005	85,127	216	61	57	4	**12.3**
NR_LBD	nuclear receptor ligand binding domain	cd06157	22,232	162	36	0	36	**10.5**
PH	PH domain	cd00900	112,411	89	72	37	35	0.0
PKc	Protein Kinase catalytic domain	cd13968	63,258	136	251	23	228	**42.9**
PPBP	Type 2 periplasmic binding fold	cd00648	54,542	177	173	151	22	**24.1**
PRTaseII	Phosphoribosyltransferase type II	cd00516	54,709	255	22	14	8	0.5
PTP	Cys, tyr & dual-specificity phosphatases	cd14494	93,503	147	52	43	9	**5.7**
PeptidS8S53	S8/S53 Peptidases	cd00306	147,746	241	40	2	38	**8.8**
RHOD	Rhodanese Homology Domain	cd00158	232,630	92	63	41	22	1.7
RPA2_OB	RPA2 oligonucleotide binding (OB) domain	cd03524	98,750	75	21	10	11	0.0
SORTASE	Sortase domain	cd00004	39,613	130	23	9	14	0.4
ZnMc	Zinc-dependent metalloprotease	cd00203	83,642	150	27	27	0	**7.8**
tRNA_Synth	Class II tRNA aminoacyl synthetases	cd00768	292,935	216	58	55	3	**12.6**
	Used for Simulations only[c]:							
PTS	Phosphotransferase system domain	cd00133	32,419	83	13	10	3	1.0
Peptidase_C19	Peptidase C19 ubiquitinyl hydrolases	cd02257	49,966	292	13	0	13	**3.6**
DNA_BREc	DNA breaking-rejoining catalytic domain	cd00397	277,438	167	11	0	11	**3.01**
FRIZZLED	Fz (frizzled) domain	cd07066	13,902	117	8	3	5	0.1
LPLAT	Lysophospholipid acyltransferases	cd06551	174,406	179	4	0	4	0.9
PARB	ParB N-terminal & sulfiredoxin domains	cd16387	66,689	56	9	7	2	0.7
RMS	Restriction-modification target recognition	cd16961	108,907	178	10	2	8	0.96
UDG_LIKE	uracil-DNA glycosylases	cd09593	51,926	131	15	4	11	0.9
ZnMP	Peptidases M48 and M56	cd05843	99,585	147	5	0	5	1.2

[a] Number of columns in CDD MSA.
[b] PDB structures sharing ≤ 65% sequence identity.
[c] At least 18 structures are required to reach significance at the p = 0.00001 level.
